# Effects of a Diabetes Prevention Program on Type 2 Diabetes Risk Factors and Quality of Life Among Latino Youths With Prediabetes: A Randomized Clinical Trial

**DOI:** 10.1001/jamanetworkopen.2022.31196

**Published:** 2022-09-12

**Authors:** Armando Peña, Micah L. Olson, Elva Hooker, Stephanie L. Ayers, Felipe González Castro, Donald L. Patrick, Libby Corral, Elvia Lish, William C. Knowler, Gabriel Q. Shaibi

**Affiliations:** 1 Center for Health Promotion and Disease Prevention, Arizona State University, Phoenix; 2 Division of Pediatric Endocrinology and Diabetes, Phoenix Children’s Hospital, Phoenix, Arizona; 3 Ivy Center for Family Wellness, The Society of St Vincent de Paul, Phoenix, Arizona; 4 Southwest Interdisciplinary Research Center, Arizona State University, Phoenix; 5 School of Public Health, University of Washington, Seattle; 6 Valley of the Sun YMCA, Phoenix, Arizona; 7 National Institute of Diabetes and Digestive and Kidney Diseases, Phoenix, Arizona

## Abstract

**Importance:**

Latino youths are disproportionately impacted by prediabetes and type 2 diabetes (T2D). Lifestyle intervention is the first-line approach for preventing or delaying T2D among adults with prediabetes.

**Objective:**

To assess the efficacy of a diabetes prevention program among Latino youths aged 12 to 16 years with prediabetes.

**Design, Setting, and Participants:**

This 2-group parallel randomized clinical trial with 2:1 randomization assessed a lifestyle intervention against usual care among Latino youths with prediabetes and obesity with 6- and 12-month follow-up. The study was conducted at YMCA facilities in Phoenix, Arizona from May 2016 to March 2020.

**Intervention:**

Participants were randomized to lifestyle intervention (INT) or usual care control (UCC). The 6-month INT included 1 d/wk of nutrition and health education and 3 d/wk of physical activity. UCC included 2 visits with a pediatric endocrinologist and a bilingual, bicultural registered dietitian to discuss diabetes risks and healthy lifestyle changes.

**Main Outcomes and Measures:**

Insulin sensitivity, glucose tolerance, and weight-specific quality of life (YQOL-W) at 6- and 12-month follow-up.

**Results:**

A total of 117 Latino youths (mean [SD] age, 14 [1] years; 47 [40.1%] girls) were included in the analysis. Overall, 79 were randomized to INT and 38 to UCC. At 6 months, the INT led to significant decreases in mean (SE) 2-hour glucose (baseline: 144 [3] mg/dL; 6 months: 132 [3] mg/dL; *P* = .002) and increases in mean (SE) insulin sensitivity (baseline: 1.9 [0.2]; 6 months: 2.6 [0.3]; *P* = .001) and YQOL-W (baseline: 75 [2]; 6 months: 80 [2]; *P* = .006), but these changes were not significantly different from UCC (2-hour glucose: mean difference, −7.2 mg/dL; 95% CI, −19.7 to 5.3 mg/dL; *P *for interaction = .26; insulin sensitivity: mean difference, 0.1; 95% CI, −0.7 to 0.9; *P *for interaction = .79; YQOL-W: mean difference, 6.3; 95% CI, −1.1 to 13.7; *P *for interaction = .10, respectively). Both INT (mean [SE], −15 mg/dL [4.9]; *P* = .002) and UCC (mean [SE], −15 mg/dL [5.4]; *P* = .005) had significant 12-month reductions in 2-hour glucose that did not differ significantly from each other (mean difference, −0.3; 95% CI, −14.5 to 14.1 mg/dL; *P *for interaction = .97). At 12 months, changes in mean (SE) insulin sensitivity in INT (baseline: 1.9 [0.2]; 12 months: 2.3 [0.2]; *P* = .06) and UCC (baseline: 1.9 [0.3]; 12 months: 2.0 [0.2]; *P* = .70) were not significantly different (mean difference, 0.3; 95% CI, −0.4 to 1.0; *P *for interaction = .37). At 12 months, YQOL-W was significantly increased in INT (basline: 75 [2]; 12 months: 82 [2]; *P* < .001) vs UCC (mean difference, 8.5; 95% CI, 0.8 to 16.2; *P *for interaction = .03).

**Conclusions and Relevance:**

In this randomized clinical trial, both INT and UCC led to similar changes in T2D risk factors among Latino youths with prediabetes; however, YQOL-W was improved in INT compared with UCC. Diabetes prevention interventions that are effective in adults also appeared to be effective in high risk youths.

**Trial Registration:**

ClinicalTrials.gov Identifier: NCT02615353

## Key Points

**Question:**

What is the efficacy of a diabetes prevention program among Latino youths with prediabetes and obesity compared with usual care?

**Findings:**

In this randomized clinical trial with 117 Latino youths, both lifestyle intervention and usual care led to significant improvements in glucose tolerance. However, lifestyle intervention significantly improved quality of life compared with usual care.

**Meaning:**

These findings suggest that increasing access to diabetes prevention services among high-risk youths may lead to reductions in type 2 diabetes rates in underserved populations.

## Teaser

This randomized clinical trial assesses the efficacy of a diabetes prevention program for improving insulin sensitivity, glucose tolerance, and quality of life among Latino youths with prediabetes.

## Introduction

Latino youths are disproportionately affected by type 2 diabetes (T2D),^1^ and these disparities emerge early in life.^2^ The US Centers for Disease Control and Prevention (CDC) estimated that Latino youths have a 50% lifetime risk of developing T2D,^3^ a preventable disease that when diagnosed in childhood reduces life expectancy^4^ and impairs quality of life (QOL).^5^ Based on compelling findings from the Diabetes Prevention Program (DPP),^6^ lifestyle intervention is considered the first-line approach for preventing T2D among adults with prediabetes.^7^ The DPP has been adapted for many high-risk adult populations,^8^ yet very few studies describe adaptations for high-risk pediatric populations.^9,10,11^

Given that Latino youths exhibit disproportionately higher rates of prediabetes compared with non-Hispanic White youths,^12^ there is a need for DPP adaptations that meet the specific needs of this underrepresented ethnic group.^13^ Adaptations that include cultural tailoring strategies are key to the development of effective diabetes prevention interventions among Latino youths.^14^ Therefore, the purpose of this study was to test the efficacy of a culturally grounded, community-based lifestyle intervention compared with usual care among Latino youths with prediabetes and obesity.

## Methods

### Participants

Participants who met the following inclusion criteria were enrolled: (1) self-reported Latino descent, (2) age 12 to 16 years, (3) body mass index (BMI) at or higher than the 95th percentile for age and sex using CDC growth charts, and (4) prediabetes as defined by a hemoglobin A_1c_ (HbA_1c_) level of 5.7% to 6.4% (to convert to proportion of hemoglobin, multiply by 0.01), fasting glucose concentration of 100 to 125 mg/dL (to convert to millimoles per liter, multiply by 0.0555), or 2-hour glucose concentration of 120 to 199 mg/dL following a 75-g oral glucose tolerance test (OGTT).^15^ Youths were excluded if they (1) were taking medication(s) or diagnosed with a condition that influences glucose metabolism, physical activity, and/or cognition; (2) met criteria for T2D (fasting glucose ≥126 mg/dL; HbA_1c_ ≥6.5%; or 2-hour glucose ≥200 mg/dL); (3) had been hospitalized within previous 2 months; (4) were enrolled in a formal weight loss program currently or within 6 months, or (5) had an uncontrolled mental health condition. This randomized clinical trial was approved by the Arizona State University (ASU) institutional review board, is in accordance with the Declaration of Helsinki,^16^ and follows the Consolidated Standards of Reporting Trials (CONSORT) reporting guidelines for trial studies.^17^ Youths provided written assent; parents provided written consent prior to study participation. Recruitment commenced in May 2016, and the study was completed in March 2020. The study protocol appears in Supplement 1.

### Study Design

This study was a 2-group parallel randomized clinical trial comparing a 6-month lifestyle intervention (INT) with a usual care control (UCC) condition.^18^ Data were collected at baseline (T1), 6 months (T2), and 1 year (T3). After T1, youths were randomized in blocks of masked size using a 2:1 ratio (INT:UCC) with the automated random sample function in SPSS statistical software version 27.0 (IBM Corp). Neither staff nor participants could determine the assigned treatment group prior to randomization. Once randomized, it was not possible to mask each participant’s treatment group, but outcome assessors were masked.

### Recruitment

Participants were recruited through local schools, community organizations, churches, and media outlets tailored to the local Latino community.^19^ Bilingual, bicultural research personnel conducted an initial telephone screening with interested individuals to confirm age, Latino descent, and BMI estimates and to provide a description of the study. Interested individuals were scheduled for a screening visit to determine eligibility.

### Eligibility Screening

Participants arrived at the ASU clinical research unit after an overnight fast. Height was measured to the nearest 0.1 cm using a portable stadiometer (SECA 213 [SECA North America]), and weight was measured to the nearest 0.1 kg using an electronic scale (TBF300A [Tanita Corporation of America]). BMI and BMI percentiles were calculated. To screen for prediabetes eligibility, blood samples were collected and analyzed by a Clinical Laboratory Improvement Amendments–certified laboratory for HbA_1c_ level, fasting glucose, and 2-hour glucose concentrations from a 75-g OGTT. Eligible participants were scheduled for their T1 visit within 4 weeks.

### T1 to T3 Study Visits

Participants returned to the clinical research unit following an overnight fast for assessment of weight-specific QOL (YQOL-W), height, weight, waist circumference, resting heart rate, seated blood pressure, and total body composition. Insulin sensitivity and glucose tolerance were measured via a multiple sample 2-hour OGTT with glucose and insulin concentrations measured at fasting and every 30 minutes. Blood samples collected for measurement of glucose (plasma) and insulin (serum) at T1 through T3 were stored at −80 °C and analyzed in batches by research laboratories at the end of the study. Physical activity was measured by the 3-day physical activity recall questionnaire.^20^ Ethnicity, country of origin, and preferred language were reported by youths. Monthly income and participation in government assistance programs were reported by parents using staff-generated questionnaires.

### Primary Outcomes

Primary outcomes included glucose tolerance, measured by 2-hour postchallenge glucose concentrations, and insulin sensitivity, estimated by the whole-body insulin sensitivity index (WBISI). WBISI was generated from insulin and glucose concentrations during the OGTT and has been validated among youths with obesity.^21^ Fasting and 30-, 60-, 90-, and 120-minute insulin and glucose concentrations were inserted into a formula (10 000 / √Fasting Glucose × Fasting Insulin × Mean OGTT Glucose × Mean OGTT Insulin) to generate a score (range, 0-12) in which higher values correspond to increased insulin sensitivity. YQOL-W was assessed by a 26-item questionnaire that considers domains of self, social, and environment as they pertain to weight-related perceptions.^22,23^ The prompts in the YQOL-W questionnaire operate on a Likert scale; examples include “Because of my weight, I avoid being seen in a swimsuit” or “Because of my weight, I am embarrassed to exercise around others.” The YQOL-W has a range of 0 to 100 in which higher scores correspond to higher levels of quality of life.

### Secondary Outcomes

Insulin secretion was estimated by the insulinogenic index (IGI), which is calculated as follows: (insulin at 30 minutes − insulin at 0 minutes) / (glucose at 30 minutes − glucose at 0 minutes). β-cell function was estimated by the oral disposition index (oDI), calculated as WBISI × IGI.^24^ Total body composition (total fat mass, total lean mass, and body fat percentage) was assessed by dual x-ray absorptiometry (Lunar iDXA [GE Healthcare]).

### INT

The INT included 1 d/wk of nutrition and health education with behavior change skills training and 3 d/wk of physical activity. Health education sessions were delivered by bilingual, bicultural community health educators from a local community clinic to groups of 8 to 10 families and promoted the adoption of a healthy balanced diet, including reducing intake of saturated fat, added sugars, and sugar-sweetened beverages, and managing portion sizes while increasing intake of fiber, fruit, and vegetables. Participants set weekly individual health behavior goals using the Specific, Measurable, Attainable, Relevant, and Timely (SMART) goal framework. Family discussions included identifying roles for eating and meal preparation at home, discussing family meals, and practicing mindfulness and respect of one another. Physical activity was delivered by YMCA instructors twice per week (60 min/session). Physical activity curriculum included circuit training, sports activities (eg, basketball, soccer), and agility and cardiovascular exercises so that average target heart rates per session were at least 150 beats per minute. To allow flexibility, a third day of unsupervised physical activity (at YMCA or off-site) was promoted by instructors to complete a minimum of 180 minutes of moderate-to-vigorous physical activity per week.

### UCC

Participants randomized to UCC met with a pediatric endocrinologist and a bilingual, bicultural registered dietitian to discuss laboratory results and develop SMART goals for making healthy lifestyle changes. These visits followed T1 and T2 laboratory visits. The UCC group was offered an abridged version of the lifestyle intervention after their T3 visit.

### Statistical Analysis

Sample size was determined using data from a previous lifestyle intervention among Latino youths with prediabetes and obesity in which we observed an effect size of *d* = 1.25 for reductions in 2-hour glucose concentrations.^25^ Using these data and assuming α = .05 and power of 85% to detect intervention effects of *d* = 1.25 on changes in glucose tolerance, a sample size of 100 was required.

Baseline characteristics between groups were compared using independent *t* tests (continuous variables) and χ^2^ tests (categorical). Changes in primary and secondary outcomes were compared between groups using latent difference score models which assess the difference in changes from T1 to T2 and T1 to T3.^26^ To avoid listwise deletion and maximize available data, we used the full-information maximum likelihood (FIML) estimation to account for missing data. The attrition in these data were 21% at T2 and 37% at T3. Using the FIML estimation ensured that all cases with valid data at baseline contributed to the estimates of intervention effects over time. Therefore, FIML accounted and adjusted for attrition over time, allowing for intent-to-treat analyses. Using FIML with a high level of missingness (eg, 50% attrition) has been shown to produce unbiased parameter estimates.^27^ Data are presented as mean (SD), FIML-adjusted mean (SE), or FIML-adjusted mean difference (95% CI) where appropriate. Data analysis was conducted in SPSS statistical software version 27.0 (IBM Corp) and MPlus version 8.7 (Muthén & Muthén).

## Results

**Figure 1.**
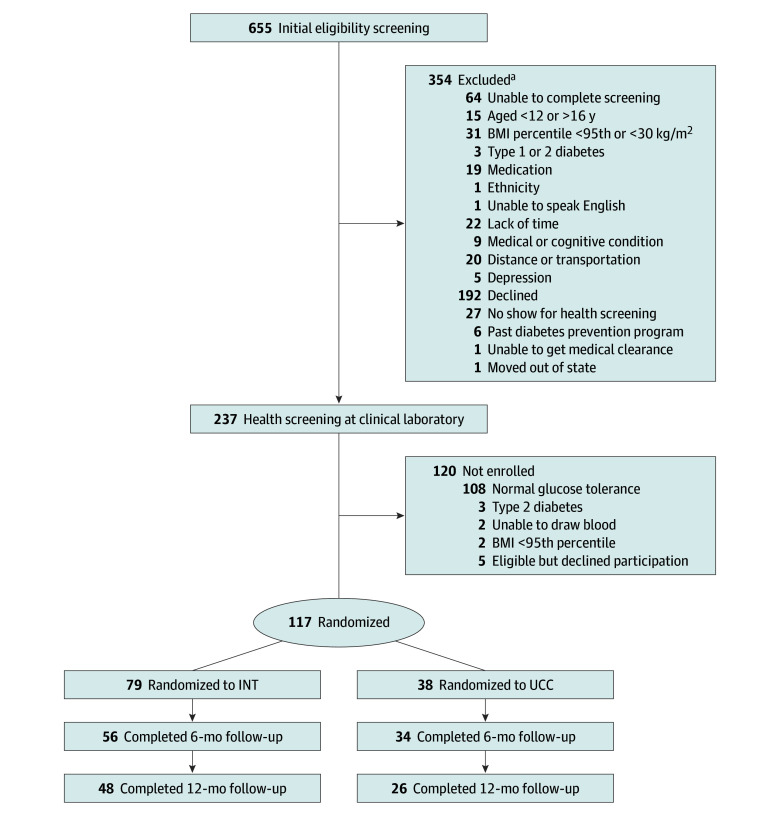
Study Flow Diagram BMI indicates body mass index; INT, lifestyle intervention; UCC, usual care control. ^a^Participants could be excluded for more than 1 reason.

Overall, 117 youths (mean [SD] age, 14 [1] years; 47 [40%] girls; mean [SD] BMI *z* score, 2.3 [0.3]) were enrolled (Figure 1). Youth met prediabetes inclusion criteria by HbA_1c_ level (30 participants), fasting glucose level (1 participant), 2-hour glucose level (57 participants), or some combination of more than 1 criteria (29 participants). Their sociodemographic characteristics are described by group in eTables 1 and 2 in Supplement 2. As a whole, nearly all were of Mexican ancestry (109 [93%]) and born in the United States. Most (96 [82%]) reported English as their preferred language. Most families (78 [67%]) reported a monthly household income from $501 to $3000. Three-fourths of families reported having Medicaid health insurance (88 [75%]), and just over one-third (44 [38%]) reported receiving the supplemental nutritional assistance program. Baseline characteristic comparisons between the INT and UCC groups are presented in Table 1, showing that groups were similar in diabetes risk factors and QOL.

**Table 1.** Baseline Characteristics

Parameter^a^	Mean (SD)		
	All (n = 117)	UCC (n = 38)	INT (n = 79)
Age, y	14 (1)	14 (2)	14 (1)
Girls, No. (%)	47 (40.1)	14 (37)	33 (42)
Boys, No. (%)	70 (60)	24 (63)	46 (58)
Height, cm	164 (9)	164 (8)	164 (9)
Weight, kg	91 (20)	95 (24)	90 (18)
Pubertal development scale, No. (%)			
Prepubertal	17 (17)	7 (21)	10 (15)
Midpubertal	51 (51)	18 (53)	33 (49)
Postpubertal	33 (33)	9 (27)	24 (36)
Gestational diabetes, No. (%)^b^	13 (12)	6 (16)	7 (10)
Family history of T2D, No. (%)			
Parents, siblings only	25 (21)	7 (18)	18 (23)
Parents, siblings, grandparents	98 (84)	30 (79)	68 (86)
BMI	34 (5)	35 (7)	33 (5)
BMI percentile	98 (1)	98 (1)	98 (1)
BMI *z* score	2 (0.3)	2 (0.4)	2 (0.3)
Waist circumference, cm	107 (14)	110 (16)	106 (13)
Fat mass	40 (12)	43 (15)	39 (11)
Lean mass	44 (9)	44 (10)	44 (9)
HbA_1c_ level, %	5.6 (0.3)	5.6 (0.3)	5.6 (0.3)
Fasting glucose, mg/dL	102 (8)	103 (7)	101 (8)
2-h Glucose, mg/dL	144 (30)	144 (29)	144 (30)
Fasting insulin, μIU/mL	24 (14)	23 (11)	24 (15)
2-h Insulin, μIU/mL	216 (176)	210 (166)	219 (181)
WBISI	2 (2)	2 (2)	2 (1)
Generic quality of life	79 (13)	75 (16)	81 (11)
Weight-specific quality of life	75 (19)	74 (18)	75 (19)

Abbreviations: BMI, body mass index (calculated as weight in kilograms divided by height in meters squared); HbA_1c_, hemoglobin A_1c_; INT, lifestyle intervention; T2D, type 2 diabetes; UCC, usual care control; WBISI, whole-body insulin sensitivity index.

SI conversion factors: To convert glucose to millimoles per liter, multiply by 0.0555; HbA_1c_ to proportion of total hemoglobin, multiply by 0.01; insulin to picomoles per liter, multiply by 6.945.

^a^ Laboratory measurements were taken at the baseline visit, not the screening visit determining eligibility.

^b^ History of gestational diabetes in the participant’s mother.

**Figure 2.**
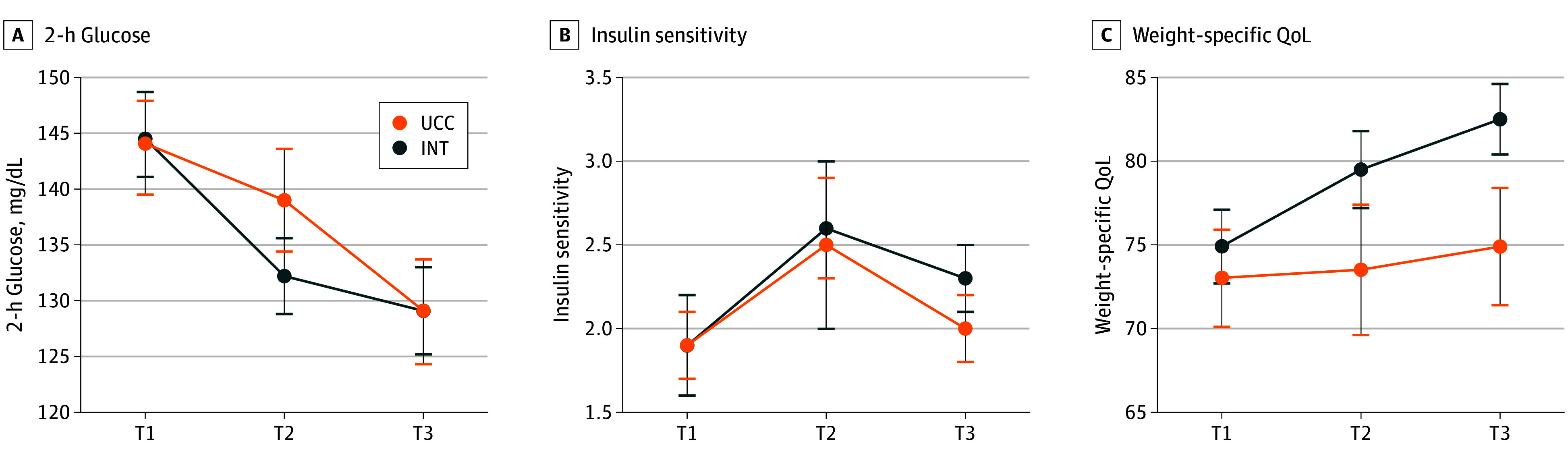
Changes in 2-Hour Glucose, Insulin Sensitivity, and Weight-Specific Quality of Life (QoL) Between Lifestyle Intervention (INT) and Usual Care Control (UCC) T1 indicates baseline; T2, 6 months; and T3, 12 months. To convert glucose to millimoles per liter, multiply by 0.0555; insulin to picomoles per liter, multiply by 6.945.

Nine youths (8 in INT, 1 in UCC) dropped out of the study for the following reasons: lack of interest (4 youths), other commitments (3 youths), or time conflicts (2 youths). Median attendance was 63% (IQR, 30%-75%) for nutrition classes and 75% (IQR, 25%-88%) for physical activity classes, while 28 youths (74%) attended both usual care visits. Changes in primary outcomes are illustrated in Figure 2 and presented in Table 2. At 6 months, mean (SE) 2-hour glucose was significantly reduced in the INT group (−12 mg/dL [5.0]; *P* = .002) but not in the UCC group (−5 mg/dL [4.0]; *P* = .31). However, the difference in changes in 2-hour glucose between groups was not significant (mean difference, −7.2 [95% CI, −19.7 to 5.3] mg/dL; *P* = .26). At 12 months, both INT (mean [SE], −15 mg/dL [4.9]; *P* = .002) and UCC (mean [SE], −15 [5.4] mg/dL; *P* = .005) groups had significant reductions in 2-hour glucose with no differences between groups (mean difference, −0.3 [95% CI, −14.5 to 14.1] mg/dL; *P* = .97).

**Table 2.** Changes in Primary and Secondary Outcomes Within and Between Usual Care Control and Lifestyle Intervention Groups From T1 to T2 and T1 to T3

Parameter	Usual care control				*P* value for within-group change		Lifestyle intervention				*P* value for within-group change		Between-group treatment effects^a^			
	No.	Mean (SE)			T2-T1	T3-T1	No.	Mean (SE)			T2-T1	T3-T1	Mean difference, T2 − T1 (95%CI)	*P* value	Mean difference, T3 − T1 (95%CI)	*P* value
		T1	T2	T3				T1	T2	T3						
2-h Glucose, mg/dL	38	144 (5)	139 (5)	129 (5)	.31	.005	78	144 (3)	132 (3)	129 (4)	.002	.002	−7.2 (−19.7 to 5.3)	.26	−0.3 (−14.5 to 14.1)	.97
WBISI	38	1.9 (0.3)	2.5 (0.5)	2.0 (0.2)	.09	.70	77	1.9 (0.2)	2.6 (0.3)	2.3 (0.2)	.001	.06	0.1 (−0.7 to 0.9)	.79	0.3 (−0.4 to 1.0)	.38
YQOL-W	38	75 (3)	73 (4)	74 (4)	.67	.78	78	75 (2)	80 (2)	82 (4)	.006	<.001	6.3 (−1.1 to 13.7)	.10	8.5 (0.8 to 16.2)	.03
Fasting glucose, mg/dL	38	103 (1)	106 (4)	102 (1)	.46	.32	79	101 (1)	99 (1)	101 (1)	.04	.83	−4.7 (−12.2 to 2.8)	.22	0.8 (−1.8 to 3.4)	.53
HbA_1c_, %	38	5.6 (0.04)	5.7 (0.10)	5.6 (0.05)	.42	.94	79	5.6 (0.03)	5.6 (0.03)	5.6 (0.04)	.37	.05	−0.06 (−0.16 to 0.04)	.23	−0.08 (−0.28 to 0.12)	.22
Weight, kg	38	95 (4)	98 (4)	101 (4)	<.001	<.001	79	90 (2)	92 (2)	94 (2)	<.001	<.001	−0.8 (−2.4 to 0.8)	.34	−1.6 (−4.4 to 1.2)	.27
BMI	38	35 (1)	35 (1)	36 (1)	.03	.004	79	33 (1)	34 (1)	34 (1)	.21	.11	−0.2 (−0.7 to 0.3)	.42	−0.5 (−1.4 to 0.4)	.27
BMI *z *score	38	2.33 (0.10)	2.32 (0.07)	2.33 (0.07)	.84	.83	79	2.25 (0.03)	2.23 (0.04)	2.20 (0.05)	.11	.05	−0.02 (−0.07 to 0.03)	.41	−0.06 (−0.14 to 0.02)	.14
WC, cm	38	110 (3)	111 (3)	113 (3)	.09	.001	79	106 (1)	107 (2)	108 (2)	.17	.07	−0.8 (−3.0 to 1.4)	.47	−1.7 (−4.4 to 1.0)	.21
Fat mass	38	43 (2)	44 (2)	45 (2)	.16	.02	79	39 (1)	39 (1)	40 (2)	.52	.11	'-1.0 (−1.5 to 0.7)	.13	−0.8 (−2.8 to 1.2)	.42
Lean mass	38	44 (2)	46 (2)	48 (2)	<.001	<.001	79	44 (1)	46 (1)	47 (1)	<.001	<.001	0.4 (−0.4 to 1.3)	.32	−0.9 (−1.4 to 0.7)	.18
Body fat, %	38	47 (1)	47 (1)	47 (1)	.21	.06	79	45 (1)	44 (1)	44 (1)	<.001	<.001	−1.0 (−1.9 to −0.04)	.04	−0.4 (−1.6 to 0.8)	.57
Fasting insulin, μIU/mL	38	23 (2)	21 (2)	22 (2)	.32	.40	78	24 (2)	19 (1)	20 (1)	.003	.01	−3.1 (−7.7 to 1.5)	.19	−3.4 (−7.4 to 0.6)	.10
IGI	38	3.3 (0.3)	3.2 (0.5)	3.2 (0.5)	.99	.81	78	3.3 (0.3)	3.1 (0.3)	3.7 (0.5)	.39	.33	−0.2 (−1.0 to 0.6)	.96	0.5 (−0.8 to 1.8)	.51
oDI	38	4.5 (0.4)	4.4 (0.4)	5.4 (0.7)	.93	.14	77	4.5 (0.3)	5.5 (0.4)	6.2 (0.6)	.04	.005	1.0 (−0.2 to 2.2)	.10	1.0 (−0.9 to 2.4)	.36
Resting HR, bpm	37	85 (2)	82 (2)	78 (2)	.09	.001	78	82 (2)	77 (1)	76 (2)	.001	.001	−2.2 (−7.1 to 2.7)	.37	0.8 (−4.5 to 6.1)	.76
SBP percentile	38	71 (5)	56 (5)	59 (6)	.001	.03	79	62 (3)	51 (4)	58 (4)	.001	.36	3.0 (−8.4 to 14.4)	.60	8.0 (−5.3 to 21.3)	.24
DBP percentile	38	71 (3)	65 (4)	72 (4)	.09	.83	79	64 (2)	66 (3)	66 (3)	.62	.64	8.0 (−1.4 to 17.4)	.10	1.0 (−7.8 to 9.8)	.83

Abbreviations: BMI, body mass index (calculated as weight in kilograms divided by height in meters squared); bpm, beats per minute; DBP, diastolic blood pressure; HR, heart rate; IGI, insulinogenic index; oDI, oral disposition index; SBP, systolic blood pressure; T1, baseline; T2, 6 months; T3, 12 months; WBISI, whole-body insulin sensitivity index; WC, waist circumference; YQOL-W, weight-specific quality of life.

SI conversion factors: To convert glucose to millimoles per liter, multiply by 0.0555; HbA_1c_ to proportion of total hemoglobin, multiply by 0.01; insulin to picomoles per liter, multiply by 6.945.

^a^ Treatment effects column displays the difference in changes in each outcome between the usual care control and lifestyle intervention groups from T1 to T2 and T1 to T3.

Mean (SE) insulin sensitivity increased by 37% following INT (baseline: 1.9 [0.2]; 6 months: 2.6 [0.3]; *P* = .001) with similar increases after UCC (32%; baseline: 1.9 [0.3]; 6 months: 2.5 [0.5]; *P* = .09); these changes were not significantly different between groups (mean difference, 0.1 [95% CI, −0.7 to 0.9]; *P* = .79). Increases in insulin sensitivity in the INT group were attenuated at T3 (mean [SE], 2.3 [0.2]; *P* = .06), whereas the UCC group returned to baseline levels (mean [SE], 2.0 [0.2]; *P* = .70), but changes were not different between groups (mean difference, 0.3 [95% CI, −0.4 to 1.0]; *P* = .38). Controlling for puberty did not substantially alter models examining changes in insulin sensitivity. Mean (SE) YQOL-W was increased following INT (7%; baseline: 75 [2]; 6 months: 80 [2]; *P* = .006) but not in the UCC group (−3%; baseline: 75 [3]; 6 months: 73 [4]; *P* = .67), with no significant difference between groups (mean difference, 6.3 [95% CI, −1.1 to 13.7]; *P* = .10). YQOL-W continued to increase in INT by T3, and these changes were significantly different than UCC (mean difference, 8.5 [95% CI, 0.8 to 16.2]; *P* = .03).

Changes in secondary outcomes are also presented in Table 2. No significant differences between INT and UCC were noted for weight, BMI, or BMI *z* score in the short term (weight: mean difference, −0.8 [95% CI, −2.4 to 0.8 kg]; *P* = .34; BMI: mean difference, −0.2 [95% CI, −0.7 to 0.3]; *P* = .42; BMI *z *score: mean difference, −0.02 [95% CI, −0.07 to 0.03]; *P* = .41) or long term (weight: mean difference, −1.6 [95% CI, −4.4 to 1.2 kg]; *P* = .27; BMI: mean difference, −0.5 [95% CI, −1.4 to 0.4]; *P* = .27; BMI *z *score: mean difference, −0.06 [95% CI, −0.14 to 0.02]; *P* = .14). Body fat percentage was significantly reduced among INT participants vs UCC participants from T1 to T2 (mean difference, −1.0 [95% CI, −1.9 to −0.04]; *P* = .04). The mean (SE) oDI significantly increased following INT at T2 (baseline: 4.5 [0.3]; 6 months: 5.5 [0.4]; 22% change; *P* = .04) and T3 (6.2 [0.6]; 38% change; *P* = .005) but these changes were not significantly different than UCC (T2: mean difference, 1.0 [95% CI, −0.2 to 2.2]; *P* = .10, T3: mean difference: 1.0 [95% CI, −0.9 to 2.4); *P* = .36). Changes in physical activity were not significantly different between groups (eTable 3 in Supplement 2).

## Discussion

Despite the increasing prevalence of T2D among children and adolescents,^2^ the evidence for diabetes prevention among youth with prediabetes remains limited.^28^ Therefore, we developed and tested a culturally grounded diabetes prevention intervention for Latino youths with prediabetes. Both INT and usual care reduced T2D risk factors to a similar degree, but YQOL-W was improved more following the INT. These findings add to the current literature focused on diabetes prevention in a vulnerable and underrepresented population subgroup.

We are aware of only 1 other randomized clinical trial to test an adapted DPP among youths with prediabetes, the Yale Bright Bodies study.^10^ Reductions in 2-hour glucose following 6 months of INT (−27 mg/dL) or standard clinical care (−10 mg/dL) were larger than in the present study.^10^ A key distinction between the Yale Bright Bodies intervention and ours was their emphasis on weight management with optional weekly weigh-ins of participants. This emphasis led to significant weight maintenance (lifestyle intervention difference, 1 kg; standard care difference, 4 kg; *P* = .006) and reduced adiposity (BMI *z* score: lifestyle intervention difference, −0.1; standard care difference, 0.04; *P* < .001) compared with the control group, whereas our study found no significant differences in weight or BMI *z* score between groups. Our curriculum emphasizes specific changes in behaviors and proximal diabetes outcomes in which participants are provided their OGTT results during intervention sessions to anchor the conversation around diabetes and health.^29^ This approach acknowledges that reductions in T2D risk factors among youths may occur in the absence of weight loss,^30^ which may be a particularly relevant strategy when tailoring diabetes prevention efforts for minority populations.^31^ Furthermore, stabilizing or maintaining weight and BMI among youths with obesity may be considered a clinically meaningful outcome.^32^

The UCC intervention that included visits with a pediatric endocrinologist and a dietitian cannot be considered a nonintervention control condition, as this level of intervention has been shown to result in reductions in 2-hour glucose.^10,33^ Many longitudinal studies, both interventional and noninterventional, have shown that in adults with impaired glucose tolerance (IGT), 2-hour glucose decreases during follow-up, even without intervention other than informing participants of their results.^6,34,35,36,37^ For example, over an average of approximately 3 years of follow-up of Pima Indian individuals with IGT, 31% progressed to diabetes, but 43% returned to normoglycemia.^35^ In the first year after randomization in the control group of the Finnish Diabetes Prevention Study clinical trial, mean 2-hour glucose declined by 5 mg/dL.^36^ In the placebo group of the US DPP, approximately 30% of those with IGT had normoglycemia after 1 year^6^; the mean decrease in 2-hour glucose in the placebo group was approximately 7 mg/dL.^37^ Similar results have been observed in at least 4 studies in children with repeat glucose tolerance tests, where IGT returned to normoglycemia in 45% of participants over 2 years in 1 study,^38^ 70% (7 of 10) returned to normal after short-term repeated tests performed over 1 to 25 days in another,^39^ 2-hour glucose declined by 10 mg/dL following standard clinical care of a clinical trial,^10^ and 62% had normoglycemia after 2 years in the fourth study.^40^ At 1-year follow-up in the present study, 59% and 50% of youths went from IGT to normoglycemia after INT or usual care, respectively, results that are comparable with the aforementioned studies. Given these variable findings on improvement in 2-hour glucose during follow-up of persons with IGT, it is not possible to determine to what extent the improvements in our study reflected treatment effects or regression to the mean.^41^ The mean 1-year changes in 2-hour glucose in the present study of 15 mg/dL in each treatment group are greater than the changes cited previously, suggesting that they may have resulted from a combination of true treatment effects and regression to the mean.

The overarching framework for this work was informed by an expanded ecodevelopmental model that considers multiple levels (ie, individual, family, community, and culture) that influence diabetes outcomes in high-risk Latino populations.^14^ To this end, our long-standing collaboration with community stakeholders informed the design of the trial that included a UCC rather than a true control condition. The UCC was intended to mirror care provided to adolescents with obesity referred to our collaborating pediatric endocrine practice. This level of care follows standard guidelines for treating pediatric obesity^42^ and prediabetes.^28^ The rationale for this approach was to consider the ethics of randomizing youth with prediabetes to a true control condition given the potential for conversion to frank T2D^38^ and to address the limited access to diabetes prevention services among low-income Latino youths in the local community among other barriers to health care.^40^

The INT was delivered in the community by our community partners who may be better positioned than researchers to implement and sustain prevention programs for high-risk populations.^43^ Given the well-established gap in translating evidence into practice,^44^ collaborations with community partners expedite the process of implementation into real-world settings and adds credibility to and expands local capacity for meeting the needs of underserved populations. Ultimately, sustaining diabetes prevention programs for high-risk youths will require empirical evidence of the intervention’s effectiveness, robust delivery systems, and advocacy for reimbursement mechanisms. This model has proven successful for scaling the DPP to a national model^45^ that is now a covered benefit for Medicare beneficiaries.^46^

In addition to increased risk of T2D, pediatric obesity is associated with reduced QOL.^47^ It is noteworthy that youths randomized to the INT exhibited increases in weight-related QOL that persisted after the intervention period. This could be a result of the social interactions inherent in a group intervention rather than a specific feature of the curriculum. A school-based weight management program among Mexican American children with overweight and/or obesity led to significant improvements in physical QOL, compared with a control group, which were accounted for by reductions in BMI *z* score.^48^ Because measures of adiposity were stable over time, changes in weight-related QOL may depend on weight reduction. As more data support a link between depression and T2D risk in adolescents,^49,50^ comprehensive interventions that integrate psychosocial health promotion within diabetes prevention are warranted to understand long-term effects.^51,52^

### Limitations

This study has limitations. The present study is the first, to our knowledge, to rigorously evaluate the efficacy of a culturally grounded lifestyle intervention aimed at preventing diabetes in Latino youths with prediabetes, but the focus on Latino youth as an underrepresented subgroup limits the generalizability to other high-risk pediatric populations. The absence of a true nonintervention control condition, which would not have been practically and ethically possible, limits the ability to estimate treatment effects definitively as both groups had similar 1-year reductions in 2-hour glucose concentrations. Additionally, the high degree of attrition that was exacerbated by the COVID-19 pandemic was handled using methods that accounted for missing data.

## Conclusions

In this study, INT led to significant short- and long-term reductions in several risk factors for T2D that were not significantly different than usual care. However, lifestyle intervention increased YQOL-W compared with usual care. How to expand T2D prevention efforts for high-risk youths with prediabetes to meet the growing demands among underserved communities warrants additional consideration.

## Notes

Supplement 1.
Trial Protocol

Supplement 2. **eTable 1.** Baseline Sociodemographic Data for All Youth **eTable 2.** Baseline Sociodemographic Data as Reported by Parents **eTable 3.** Changes in Physical Activity Within and Between Groups

Supplement 3.
Data Sharing Statement
