# Errors in Abstract and Methods

**DOI:** 10.1001/jamanetworkopen.2022.39217

**Published:** 2022-10-06

In the Original Investigation titled “Effects of a Diabetes Prevention Program on Type 2 Diabetes Risk Factors and Quality of Life Among Latino Youths With Prediabetes: A Randomized Clinical Trial,”^1^ published September 12, 2022, there were errors in the Abstract and Methods sections. In the Conclusions and Relevance section of the Abstract, the population should have been described as Latino youths with prediabetes. In the Methods section, the formula for the insulinogenic index should have read (insulin at 30 minutes − insulin at 0 minutes) / (glucose at 30 minutes − glucose at 0 minutes). This article has been corrected.^1^
