# Errors in Table 2

**DOI:** 10.1001/jamanetworkopen.2024.60689

**Published:** 2025-01-15

In the Original Investigation titled “Effects of a Diabetes Prevention Program on Type 2 Diabetes Risk Factors and Quality of Life Among Latino Youths With Prediabetes: A Randomized Clinical Trial,”^1^ published September 12, 2022, there were errors in Table 2. The column for *P* value for within-group change between baseline and 6 months in the lifestyle intervention group included incorrect *P* values for the following rows: weight (should have read <.001), body mass index (should have read .21), body mass index *z* score (should have read .11), waist circumference (should have read .17), fat mass (should have read .52), lean mass (should have read <.001), percentage body fat (should have read <.001), fasting insulin level (should have read .003), insulinogenic index (should have read .39), oral disposition index (should have read .04), resting heart rate (should have read .001), systolic blood pressure percentile (should have read .001), and diastolic blood pressure percentile (should have read .62). This article has been corrected.^1^
