# Supplementary material for: Effects of a Diabetes Prevention Program on Type 2 Diabetes Risk Factors and Quality of Life Among Latino Youths With Prediabetes: A Randomized Clinical Trial
Source: JAMA Netw Open. 2022 Sep 12;5(9):e2231196. doi: 10.1001/jamanetworkopen.2022.31196 (PMC9468887; doi:10.1001/jamanetworkopen.2022.31196)
Supplement: Supplement 1. — Trial Protocol [file jamanetwopen-e2231196-s001.pdf]

Instructions and Notes:

- Depending on the nature of what you are doing, some sections may not be applicable to your research. If so mark as "NA".
- When you write a protocol, keep an electronic copy. You will need to modify this copy when making changes.

1 Protocol Title

Diabetes Prevention for Latino Youth with Prediabetes

2 Background and Objectives

Provide the scientific or scholarly background for, rationale for, and significance of the research based on the existing literature and how will it add to existing knowledge.

- Describe the purpose, specific aims, or objectives.
- State the hypotheses to be tested.
- Describe the relevant prior experience and gaps in current knowledge.
- Describe any relevant preliminary data.

Type 2 Diabetes (T2D) is a major public health burden that reduces quality of life (QoL) and longevity. Disparities in T2D emerge early in life and disproportionately impact obese Latino youth. The CDC estimates that up to 50% of Latino children will develop T2D in their lifetime. Given that youth onset T2D is estimated to reduce life expectancy by 15 years, a public health imperative is to prevent this chronic disease in youth. The Diabetes Prevention Program (DPP) established that T2D is preventable through lifestyle intervention in adults with prediabetes, yet to date, no T2D prevention studies for prediabetic Latino youth exist in the literature.

Intensive and targeted T2D prevention efforts for youth who are already obese and exhibit prediabetes are critical, as this group is at imminent risk for conversion to T2D and the associated complications. In prediabetic adults, weight loss is the primary factor mediating T2D prevention. In contrast we have shown, through a series of lifestyle interventions in obese Latino youth, that significant health improvements occur independent of weight loss. Since obese youth are still developing, improvements in body composition may be more physiologically relevant for T2D prevention. Therefore, we propose that weight loss may not be the optimal target for preventing T2D in obese prediabetic youth. This paradigm acknowledges that 1) most obese adolescents will remain obese for the rest of their lives, 2) improving metabolic health in obese adolescents is not necessarily linked to weight loss, and 3) intermediate T2D outcomes (e.g., glucose tolerance and insulin sensitivity) are needed to appreciate T2D risk reduction in response to intervention. In addition, reframing intervention goals and outcomes around improved health and well-being rather than weight loss per se will further enhance program compliance, retention, and ultimately success among obese youth.

Challenges to implementing effective diabetes prevention programs for Latino youth with prediabetes include disparities in access to care, cultural norms surrounding obesity and diabetes, and poor health literacy in low income, Spanish-speaking families. Unfortunately, the current healthcare system is poorly equipped to address these contextual factors and meet diabetes prevention needs of obese Latino youth with prediabetes.

Our transdisciplinary team of researchers, practitioners, and community partners has collaborated for the past 7-years to effectively address diabetes prevention challenges through the development, testing, refinement, and expansion of a culturally-grounded lifestyle intervention for obese Latino youth with prediabetes, which if efficacious, could be taken to scale. The overall approach applies an adapted Ecodevelopmental framework to leverage community, family, peer, and individual-level influences during the critical transition period of adolescence when changes in health behaviors and health outcomes are linked to future health trajectories. The intervention draws upon Social Cognitive Theory and employs key behavioral modification strategies from the DPP to enhance self-efficacy and foster social support for making healthy behavior changes. *Promotores* (community health workers) deliver nutrition education to families to facilitate dietary changes while group exercise classes for youth are led by YMCA staff to increase physical activity levels. Feasibility and initial efficacy for improving glucose tolerance and increasing insulin sensitivity was established through a pilot study. The pilot intervention also led to significant improvements in QoL, eating behaviors, fitness, and multiple cardiovascular disease risk factors without weight loss. We are currently conducting an RCT that expands upon the pilot to rigorously evaluate intervention effects in obese Latino adolescents. To date, we have enrolled 160 obese youth and the rate of prediabetes in this community sample is ~15%. Preliminary analyses on prediabetic youth show robust and sustained improvements on targeted outcomes including glucose tolerance, insulin sensitivity, and QoL. For ethical reasons, we do not randomize prediabetic youth to the control condition in that study. We now propose to build upon these compelling preliminary findings and further refine our culturally-grounded lifestyle intervention to test the efficacy, as compared to usual care, for improving health and QoL by **preventing T2D in obese Latino adolescents with prediabetes**.

Specific Aim 1: Test the short-term (6-month) and long-term (12-month) efficacy of this culturally-grounded, community-based lifestyle intervention as compared with a usual care control group for improving glucose tolerance and increasing insulin sensitivity in 120 obese Latino adolescents age 12-16 with prediabetes.

Specific Aim 2: Compare intervention effects to usual care on changes in QoL.

Specific Aim 3: Explore the mediating effects of changes in body composition on changes in T2D risk markers.

Specific Aim 4: Estimate the incremental cost effectiveness for improving glucose tolerance between groups.

3 Data Use

Describe how the data will be used. Examples include:

- Dissertation, Thesis, Undergraduate honors project
- Publication/journal article, conferences/presentations
- Results released to agency or organization
- Results released to participants/parents
- Results released to employer or school
- Other (describe)

- **Data will be used in dissertation, thesis, and undergraduate honors projects.**
- **Data will be used for publication/journal article, conferences/presentations.**
- **Results may be released to agencies or organizations.**
- **Results may be released to participants.**

4 Inclusion and Exclusion Criteria

Describe the inclusion and the exclusion criteria for the study.
Describe how individuals will be screened for eligibility.
Indicate specifically whether you will target or exclude each of the following special populations:

- Minors (individuals who are under the age of 18)
- Adults who are unable to consent
- Pregnant women
- Prisoners
- Native Americans
- Undocumented individuals

Inclusion Criteria:

- Latino: self-report
- Age: 12-16
- Obese: BMI percentile ≥95th percentile for age and gender or BMI ≥30 kg/m^2
- Prediabetic: fasting glucose ≥100 and/or 2-hour post-OGTT glucose ≥120 mg/dl

Exclusion Criteria:

- Taking medication(s) or diagnosed with a condition that influences carbohydrate metabolism, PA, and/or cognition
- Type 2 diabetes: Fasting glucose ≥126 mg/dl or 2-hour glucose ≥200 mg/dl.
  - Youth found to be diabetic upon screening will be referred for follow-up care
- Recent Hospitalization (previous 2 months)
- Currently enrolled in (or within previous 6 months) a formal weight loss program.
- Diagnosed depression or other condition that may impact QoL
- Pregnant

5 Number of Participants

Indicate the total number of participants to be recruited and enrolled

- Provide a rationale for the proposed enrollment number
- What percentage of screened individuals will likely qualify for the study?

We will screen ~300 obese Latino youth for prediabetes and anticipate that 120 will meet eligibility into the trial. These estimates are based on rates of elevated 2-hour glucose in our current study (~40% exhibit 2-hour glucose ≥120 mg/dl).

6 Recruitment Methods

- Describe when, where, and how potential participants will be identified and recruited.
- Describe materials that will be used to recruit participants. (Attach copies of these documents with the application.)
- Does any member have a dual role with the study population?

Recruitment will be coordinated by our clinical partner from the Family Diabetes Program at the St. Vincent de Paul Medical and Dental Clinic, an established and trusted entity in the local Latino community. The clinic provides primary and specialty services to Latino children and families and pulls from a referral network of over 100 schools, community centers, and healthcare organizations in the greater Phoenix area. This referral network is our primary source of recruitment for our ongoing RCT in a similar population where we have successfully met our recruitment schedule. Successful recruitment efforts are facilitated through distribution of flyers, hosting presentations, attending health fairs, and direct contact/referral by providers (see letters of support). Additional aspects that facilitate successful recruitment and enrollment include, 1) bilingual / bicultural research staff to assist with recruiting, consenting, enrollment and data collection, 2) pediatric and specially trained research nursing staff in our clinical unit, 3) assistance with transportation to and from research and intervention facilities, 4) appropriate compensation for time and effort, 5) A Community Advisory Board of trusted community organizations serving Latino families (see letter of support from CAB Chair), 6) providing families the results of the metabolic testing, and most importantly 7) providing an intervention with a high likelihood of directly benefiting individuals enrolled.

7 Study Timelines

Describe:

- The duration of an individual participant's participation in the study.
- The duration anticipated to enroll all study participants.
- The estimated date for the investigators to complete this study (up to and including primary analyses).

Each participant will be enrolled for ~13 months and we anticipate that it will take ~3.5 years to enroll the entire sample. We anticipate that the study will be completed in November of 2021.

8 Procedures Involved

Describe and explain the study design. Provide a description of all research procedures being performed and when they are performed. Describe procedures including:

- The documents/ measures / devices/ records /sampling that will be used to collect data about participants. (Attach all surveys, scripts, and data collection forms.)
- What data will be collected including long-term follow-up?
- All drugs and medical devices used in the research and the purpose of their use, and their regulatory approval status.
- Describe the available compensation (monetary or credit that will be provided to research participants).
- Describe any costs that participants may be responsible for because of participation in the research.

Design: This is a randomized controlled intervention to test the short-term (6-months) and long-term (12-months) efficacy of a culturally-grounded lifestyle intervention to improve glucose tolerance in obese Latino adolescents with prediabetes compared to a UCC group. We will further determine effects on QoL, explore the mediating effects of changes in total body fat on T2D risk reduction, and examine the initial incremental cost effectiveness of the intervention to improve glucose tolerance compared to usual care control.

Informed consent: Written informed consent and assent will be obtained from the parent/guardian and adolescent prior to any procedures. Participants will be informed they are free to withdraw from the study at anytime, that nonparticipation will not affect services, and that confidentiality will be maintained. All study-related documents will be approved by the ASU IRB and materials will be available in English and Spanish with bilingual/bicultural research staff administering consent, collecting data, and answering questions.

Health Screening and Metabolic Phenotyping: Potential participants will arrive at the ASU Clinical Research Unit at ~8:00 AM after an overnight fast for initial screening including a brief health history, height and weight measurement to calculate BMI percentile, and a standard 75g OGTT for fasting and 2-hour glucose (assessed by SonoraQuest Laboratories). Participants meeting inclusion criteria (described above) will return within 4 weeks of the screening OGTT for baseline testing to include in-depth medical and family history (including in utero exposure to gestational diabetes) and a brief physical exam. Height, weight, and waist circumference will be measured to the nearest 0.1cm, 0.1 kg, 0.1cm, respectively. Sitting blood pressure will be measured in triplicate using an appropriately-sized cuff on the right arm after 5 min of rest. A fasting lipid panel will be collected to measure cholesterol (Total, HDL, and LDL), triglycerides, and HbA1c. These assessments follow current recommendations for screening in obese youth, will be used in the curriculum to discuss the health risks of obesity, and are an important and meaningful benefit to families.

Glucose Tolerance and Insulin Sensitivity (Aim 1): Glucose tolerance and insulin sensitivity will be assessed by a 75 gram OGTT with multiple blood sampling for insulin glucose. Blood samples will be collected from an in-dwelling catheter at -15’, -5’, 30’, 60’, 90’ and 120’ for measurement of plasma glucose (glucose oxidase, YSI INC., Yellow Springs, OH) and insulin (ELISA, ALPCO Diagnostics, Windham, NH). At time 0’, participants will ingest 75 grams of glucose in solution. Improvements in glucose tolerance will be assessed by decreases in 2-hour glucose levels (primary outcome) while changes in insulin sensitivity will be estimated by the whole-body insulin sensitivity index. For the purposes of this study, glycemic status will be defined as “Normal” (fasting glucose <100 mg/dl and 2-hr glucose <120 mg/dl), “Prediabetic” (fasting glucose ≥100 mg/dl or 2-hr glucose ≥120 mg/dl), or “Diabetic” (fasting glucose ≥126 mg/dl or 2-hr glucose ≥200 mg/dl). NOTE: Youth identified as “diabetic” at any point in the study will be referred for follow-up care and given the option to continue with the study with clearance from their physician.

Quality of Life (Aim 2): QoL will be assessed using the Youth Quality of Life (YQOL) inventory. The YQOL was developed through semi-structured interviews with youth regarding positive and negative aspects of QoL. Domains of self (feelings about one’s self), social relationships (friends and family), environment (social and cultural milieu) are assessed and an overall QoL score is computed. Weight-specific QoL will be assessed by the YQOL-W which, measures three domains of weight-related QoL (Self, Social, and Environmental). It is specific to obese adolescents (11-18 years) for use in evaluating weight management interventions in clinical and community research. Both instruments will be self-administered prior to any anthropometric procedures and can be completed in <15 minutes.

Total Body Composition (Aim 3): Total body composition (fat, muscle, and bone) will be assessed by Dual-energy X-Ray Absorptiometry (DXA) using the GE Lunar iDXA (GE Lunar, Madison, WI). DXA provides reasonable estimates of total body composition as well as changes in fat mass and lean tissue mass following lifestyle intervention in obese youth. Total scan time will be ~ 15 minutes while the DXA arm passes over the entire body. DXA scans will be performed upon completion of the OGTT, prior to eating. A urine pregnancy test will be performed in females in the ASU Clinical Research Unit with a negative test required before each DXA measurement (baseline, 6- and 12-months). Pregnant females will be excluded.

Physical Activity, Fitness and Nutrition Assessment: PA will be measured using the 3 Day PA Recall, (3DPAR), an interviewer-administered recall instrument that measures the type of PA performed during the past 3 days (e.g. Tues, Mon, Sun). The 3DPAR allows for assessment of time spent in sedentary behaviors and types of activity that can be useful to identify differences in PA patterns between adolescents. Cardiorespiratory fitness ($Vo2_{peak}$) will be estimated by a submaximal exercise test developed and validated for obese youth. Participants walk on a treadmill at a self-selected speed at 0% grade for 4 minutes. The grade is then increased to 5% while speed is maintained for 4 more minutes. Heart rate is recorded at the end of the 8 minutes and entered into the prediction equation. Dietary intake will be measured using the 2007 Block Food Screener for Ages 2-17. This 41-item screener assesses foods eaten during the previous week and was designed to identify dietary intake by food group.[144] National dietary surveys were used to inform the food selections to query, as well as to identify appropriate portion sizes and nutrient composition. This screener includes items commonly consumed by Latino youth and has been used to assess changes in dietary factors in Latino youth.

Intervention (Nutrition Education): The intervention curriculum was developed through an inductive, collaborative process with our community partners and subsequently refined through a series of increasingly rigorous projects starting with a clinical demonstration project, a pilot study, and our ongoing RCT. The curriculum is informed by Social Cognitive Theory and applies key behavioral change strategies from the adult DPP and other successful lifestyle interventions such as goal-setting, fostering social support, and enhancing self-efficacy to facilitate health behavior change. The education curriculum is tailored for the unique psychosocial and developmental characteristics of obese adolescents and grounded in the Latino culture. Sessions (N=20) are delivered at the YMCA to groups of 8-10 families (parent/guardian participation is a requirement) by bilingual/bicultural community health educators from the Family Diabetes Program at St. Vincent de Paul Medical and Dental Clinic.

Our intervention is innovative in that it, unlike the weight loss goal of >7% in the DPP, focuses on health gains (primarily diabetes risk reduction) as the primary goal. Enhancement of nutrition and physical activity-related behaviors are enacted through the interventions critical inputs and include 1) fostering social support from family and peers and 2) enhancing self-efficacy for making healthy behavior changes. Social support is fostered through a) Appraisal: Providing OGTT results from screening and basing health goals aimed at reducing T2D risk, b) Informational: Health Educators deliver diabetes-related health and nutrition education, c) Instrumental: Families exchange contact information and children exchange school information to facilitate interaction outside of the program, and d) Emotional: Emotional well-being is woven throughout the curriculum with an emphasis placed on building self-esteem, positive self-affirmation, and reducing negative influences from family and peers. Self-efficacy is enhanced through a) Goal-setting: Setting, monitoring, and achieving health behavior goals, b) Vicarious Experience & Role Modeling: Health educators and peers role play situations and exercise staff model activities, and c) Verbal Encouragement: Health educators, family, and peers encourage youth to make healthy behavior changes.

Children are presented with the results of their glucose tolerance test in the first class as a way to frame the discussion around diabetes and health. Each session begins with a low fat, high fiber snack and recipe (role modeling), acknowledgment and reinforcement of healthy behavior changes and progress towards individual goals (emotional and instrumental support), problem-solving challenges, and an outline of the session's goals. Children are incentivized through a point system for attendance, completing out of class 'assignments' such as helping to prepare a healthy meal for the family (enacting behaviors), participating in group discussions, and making progress towards their individual health goals. Parents and children work with other families during classes and are encouraged to do so outside of class in order to build a support network that extends beyond the program. Classes are delivered using a tiered approach where the first 16-sessions are delivered weekly while the last 4 session are spread over 8 weeks (total intervention period is 6-months). We have found that this delivery approach builds resiliency within families as they become less reliant on the health educators and more independent in achieving their health and behavior goals. Following the intervention period, children will return for post-intervention testing.

Intervention (Physical Activity): The PA intervention includes structured and unstructured components. The structured PA curriculum is led by YMCA instructors and delivered 2 days/week to groups of 8-10 youth (both boys and girls) for 60-minutes each. Classes include aerobic and resistance exercises delivered in a progressive manner with the first 2-4 weeks focusing on motor skill acquisition, exercise confidence, developing a fitness base, and building camaraderie among participants. Aerobic exercises include group activity classes (e.g. spinning and cardio kick-boxing) with the goal of maintaining heart rates > 150 BPM. Real-time heart rate monitoring and rate of perceived exertion are used to monitor and document exercise intensity throughout the program. This exercise intensity was selected for the established effects on improving metabolic health in obese youth. Resistance exercise includes circuit training using age and size appropriate equipment and is incorporated because our previous studies suggest this form of exercise is both enjoyable and metabolically beneficial for obese youth. In addition to structured PA classes, youth are 'prescribed' an additional day of unstructured PA of at least 60-minutes with a family member or peer in the program. This allows for flexibility in pursuing preferred activities that can be done at the YMCA or elsewhere in the community in order to promote social support, role modeling, bonding among youth and families, and facilitate sustainability.

Booster Sessions: Three booster sessions (months 7, 8, and 9) following the completion of the intensive lifestyle period will be held to support the maintenance of healthy lifestyle behaviors, address any challenges encountered, and promote successes achieved. Post-intervention clinical measures will be returned to participants at the first session and changes in health status will be discussed in the context of maintaining healthy lifestyle behaviors.

Usual Care Control (UCC): Participants randomized to the UCC will meet with a pediatric endocrinologist (Micah Olson, MD) to review laboratory results at baseline and 6-months. In addition, UCC participants will meet with a registered dietitian (Yolanda Konopken, RD) who will provide general lifestyle counseling on healthy eating and PA at baseline and 6-months. This approach mirrors local standards/current practice for obese youth referred to weight management. Randomizing obese children with prediabetes to a true control condition is not ethical as our previous and current studies suggest rapid decompensation occurs in this group. To ensure retention in UCC participants, the research team will contact youth via phone, text, or email (youth/parent preference) on a monthly basis throughout the entire study period (months 1-12). Upon completion of the study, control youth will be offered an abridged version of the intervention and a 1-year membership to the YMCA. Although it would be preferable to offer the entire intervention to UCC youth, this is not feasible due to study costs and time constraints.

Post-intervention Follow-up: All youth, regardless of group, will be contacted on a monthly basis via phone, text, or email to enhance retention and ensure availability for testing 12-months after baseline.

Cost and Compensation: There is no cost to participate in this study. Participants will be reimbursed up to $50 for their time and travel for each clinical testing visit and will receive a 12-month YMCA membership. In addition, participants will be able to earn incentives such as T-shirts, water bottles, and gift cards for attendance, participation, and responding to retention efforts.

9 Withdrawal of Participants

Describe anticipated circumstances under which participants will be withdrawn from the research without their consent.
Describe procedures that will be followed when participants withdraw from the research, including partial withdrawal from procedures with continued data collection.

- Participants are free to withdraw from the study at any time. If a participant notifies a staff member that they no longer want to participate in the study, this choice will be documented in their study chart and in the database. They will be told that withdrawal will not impact any services or relationship with ASU, St. Vincent de Paul, or the YMCA. We will not be scheduled for any future study visits.
- The participants may be asked to leave the study if they:
  - Are disruptive or inappropriate during the intervention.
  - Do not attend at least 75% of the intervention sessions (54 of the 72 total).
- Participants will be contacted by study staff who will provide the justification for being withdrawn and answer any questions.

10 Risks to Participants

List the reasonably foreseeable risks, discomforts, hazards, or inconveniences to the participants related the participants' participation in the research. Include as may be useful for the IRB's consideration, the probability, magnitude, duration, and reversibility of the risks. Consider physical, psychological, social, legal, and economic risks. Reference this information when appropriate.

- If applicable, indicate which procedures may have risks to an embryo or fetus should the participant be or become pregnant.
- If applicable, describe risks to others who are not subjects.

- The risks include the hazards associated with fasting, blood draw, participation in physical activity (e.g., musculoskeletal injury), and exposure to radiation during the DEXA.
- Risks will be substantially reduced by the following:
  - Certified phlebotomists and pediatric registered nurses to perform the blood draws. Phlebotomists and nurses will monitor venipuncture sites for bruising or discomfort and appropriate cleaning and bandaging procedures will be followed.
  - To minimize risk associated with exercise, the physical activity program was developed by a certified fitness instructor at the YMCA that is specifically designed for obese youth ages 12-16. The program will include an adequate warm-up and cool-down period for exercise sessions and will be led by certified fitness instructors.
  - The radiation exposure during DEXA is minimal and is similar to a cross-country airplane flight (~0.03 mSv).

11 Potential Benefits to Participants

Realistically describe the potential benefits that individual subjects may experience from taking part in the research. Include the probability, magnitude, and duration of the potential benefits. Indicate if there is no direct benefit. Do not include compensation or benefits to society or others.

- There may not be a direct benefit to participants other than the knowledge gained from the health education program. We have found that similar programs result in improvements in psychosocial health, health behaviors, and health outcomes and that the reduction in T2D risk is considerable.

12 Setting

Describe the sites or locations where your research team will conduct the research.

- Identify where research procedures will be performed.
- For research conducted outside of the ASU describe:
  - Site-specific regulations or customs affecting the research.
  - Local scientific and ethical review structures in place.

- The research will take place at ASU DPC and the Downtown YMCA.
- The YMCA does not have any site-specific regulations or customs and will defer to ASU's IRB for ethical oversight. We have been partnering with the YMCA on similar projects for the past 7 years and have developed a strong collaborative relationship with the organization (See letter of support).

13 Multi-Site Research

If this is a multi-site study where you are the lead investigator, describe the processes you will use to ensure communication among sites, such as:

- Each site has the most current version of the protocol, consent document, and HIPAA authorization.
- Required approvals have been obtained at each site (including approval by the site's IRB of record).
- Describe processes you will use to communicate with participating sites.
- Participating sites will safeguard data as required by local information security policies.
- Local site investigators conduct the study appropriately.

N/A

14 Resources Available

Describe the qualifications (e.g., training, experience, oversight) of you and your staff as required to perform your roles. When applicable describe knowledge of the local study sites, culture, and society. Provide enough information to convince the IRB that you have qualified staff for the proposed research.

Describe other resources available to conduct the research: For example, as appropriate:

- Describe your facilities.
- Describe the availability of medical or psychological resources that participants might need as a result of any anticipated consequences of the human research.
- Describe your process to ensure that all persons assisting with the research are adequately informed about the protocol, the research procedures, and their duties and functions.

The PI, research team, and study staff have extensive experience in conducting collaborative, community-based health promotion and disease prevention studies using similar procedures in similar populations. The study team is comprised of bilingual/bicultural researchers and staff members with an in-depth knowledge of the local Latino community. The clinical research facilities at ASU provides an ideal venue for the proposed clinical testing procedures and we employ several pediatric nurses and phlebotomists who provide coverage on study days. The research team also includes a Pediatric Endocrinologist (Micah Olson, MD) who is an adjunct professor in the College of Nursing and will provide medical oversight. New study staff will complete comprehensive training from the PI and study coordinator that includes review of prior studies, review of the study protocol, and study manual, and shadowing of experienced staff prior to performing any procedures.

15 Prior Approvals

Describe any approvals that will be obtained prior to commencing the research. (E.g., school, external site, funding agency, laboratory, radiation safety, or biosafety approval.)

Radiation safety approval will be obtained prior to commencing the research and we will add this protocol to our current IBC disclosure # 12-491.

16 Data Management and Confidentiality

Describe the data analysis plan, including procedures for statistical analysis.

Describe the steps that will be taken to secure the data during storage, use, and transmission.

- Training, authorization of access, password protection, encryption, physical controls, certificates of confidentiality, and separation of identifiers and data

Describe how data and any specimens will be handled:

- What personal identifiers will be included in that data or associated with the specimens?
- Where and how data or specimens will be stored?
- How long the data or specimens will be stored?
- Who will have access to the data or specimens?
- Who is responsible for receipt or transmission of the data or specimens?
- How will data and specimens be transported?
- If data or specimens will be banked for future use, describe where the specimens will be stored, how long they will be stored, how the specimens will be accessed, and who will have access to the specimens.
- Describe the procedures to release data or specimens, including: the process to request a release, approvals required for release, who can obtain data or specimens, and the data to be provided with specimens.

Specific Aim 1: We will test the hypothesis that adolescents who complete the intervention will exhibit significantly greater short-term and long-term improvements in glucose tolerance and increases in insulin sensitivity compared to UCC. ANCOVA models will use pretest measures as a covariate and the experimental groups (i.e., intervention vs. UCC) for comparisons. Separate analyses will be conducted for 6- and 12-months. We will explore interactions between the covariate (baseline measure) and group to determine whether adolescents with lower baseline scores differentially benefit from the program. Assuming no interaction is found, we will examine the main effect of group assignment on the outcome measure after adjusting for baseline. We will also conduct repeated measures ANCOVA models to examine differences in the effect of the intervention on primary outcomes between 6- and 12-month time points.

Specific Aim 2: We will use the same ANCOVA models described above to examine the effects of the intervention on general and weight-specific QoL. Results will indicate whether adolescents who were randomized to the intervention report greater increases in QoL compared to adolescents randomized to UCC.

Specific Aim 3: We will explore the mediating effects of changes in total body composition on changes in T2D risk markers by path analysis. Each mediation model will include three variables: (1) experimental group membership represented by dummy codes, (2) a mediator measure (e.g., fat mass), and (3) an outcome measure. The model specifies direct paths from group membership to the mediator and outcome. Path coefficients and standard errors for these paths will be estimated. Effect size measures will include: (1) direct effects on outcome and (2) mediated or indirect effects on outcome. The latter will be estimated using path coefficient estimates with standard errors.

Specific Aim 4: To examine the cost effectiveness of the intervention, we will estimate the incremental cost-effectiveness ratio (ICER) of the intervention compared to UCC based on changes in 2-hour glucose. The cost effectiveness analyses will be conducted from the societal perspective using 2-hour glucose at 12 months, direct medical costs, and non-medical costs. Direct medical costs include: personnel, intervention materials, MD and RD visits, lab tests, and procedures. The non-medical costs will be participant time for travel, productivity loss, and commercial services for PA and nutrition. Base case and sensitivity analyses will be conducted using TreeAge software. The base case analysis model will use 6-month intervention costs and glucose tolerance at 12-months. ICERs will be calculated by dividing incremental costs by incremental effectiveness (change in 2-hour glucose). The results will indicate whether improved glucose tolerance leads to cost savings. Costs collected throughout the study will be inflated based on the published inflation rates in US dollars at the end of the study.

Confidentiality will be protected through the use of study identification numbers that will be kept separate from personal identifiers. A secure, password protected database will serve as the link between study IDs and source documents which will be kept in separate double-locked file cabinets at Arizona State University in the Southwest Interdisciplinary Research Center (SIRC) data storage in the UCENT building room 700. With the exception of the consent, contact information sheet, eligibility, clearance forms, and lab reports used in the context of the lifestyle class, all study materials will identify participants solely by the assigned code numbers. Blood specimens will be stored with ID numbers in the PI's freezer in ABC-1 rm 259-A. Members of the research team will have access to de-identified data. ID numbers will be used and de-identified data will be entered into a password protected computerized database after baseline testing and confirmation of eligibility. Data will be stored for 7 years; paper documents will be shredded and electronic files will be erased.

17 Safety Monitoring

This is required when research involves more than Minimal Risk to participants. The plan might include establishing a data monitoring committee and a plan for reporting data monitoring committee findings to the IRB and the sponsor. Describe:

- The plan to periodically evaluate the data collected regarding both harms and benefits to determine whether participants remain safe.
- What data are reviewed, including safety data, untoward events, and efficacy data?
- How the safety information will be collected (e.g., with case report forms, at study visits, by telephone calls with participants).
- Who will review the data?

In compliance with the National Institutes of Health policy for protection of human subjects in clinical studies, we will implement the following Data Safety Monitoring Board (DSMB) policy. This DSMB policy serves to protect the health and safety of human subjects and provide information relevant to subjects' continuation in clinical studies. The DSMB will include the Chair (Dr. David Coon), the Associate Dean of Research in the College of Nursing (Dr. Elizabeth Reifsnider), a statistician, and senior investigators. This DSMB will meet quarterly to review progress pursuant to presentation of a summary report from Dr. Shaibi by ensuring that policies on the identification and reporting of adverse events to the appropriate regulatory bodies, which can include the ASU institutional review board and the project officer at the National Institutes of Health, have been implemented diligently and promptly. Prompt and due diligence in reporting of adverse events (i.e., within 24 hours during the week and on the next working day following a weekend) to the regulatory bodies will remain the express duty of the PI. At baseline and every 4 weeks during data collection, we will examine the results for evidence of harm to the study participants. We will evaluate reports from participants or research personnel regarding pain, discomfort, or injury subsequent to participation in the study.

18 Consent Process

Describe the process and procedures process you will use to obtain consent. Include a description of:

- Who will be responsible for consenting participants?
- Where will the consent process take place?
- How will consent be obtained?
- If participants who do not speak English will be enrolled, describe the process to ensure that the oral and/or written information provided to those participants will be in that language. Indicate the language that will be used by those obtaining consent. Translated consent forms should be submitted after the English is approved.

- The research coordinator or a designated staff member will be responsible for obtaining written informed consent (permission from parents) and assent from participants at ASU.
- All procedures will be explained to families in person in detail (their preferred language of English or Spanish). Any questions will be addressed. Special care will be taken to explain the assent document in detail to youth to ensure comprehension. Youth will be asked to explain back to research team what the study is about and what is involved to ensure appropriate explanations. Consents will be available in English and Spanish and the research team is comprised of several bilingual/bicultural members with extensive experience working with the Latino community. After the study team has explained the purpose and procedures verbally, participants will be asked to provide the team with a brief overview of the study to ensure comprehension. Questions may include: "Can you tell me why we are doing this study? Can you tell me one of the risks? Can you tell me one of the benefits?"

19 Investigational New Drug or Devices

If the drug is investigational (has an IND) or the device has an IDE or a claim of abbreviated IDE (non-significant risk device), include the following information:

- Identify the hold of the IND/IDE/Abbreviated IDE.
- Explain procedures followed to comply with FDA sponsor requirements for the following:

	Applicable to:		
**FDA Regulation**	**IND Studies**	**IDE studies**	**Abbreviated IDE studies**
21 CFR 11	X	X	
21 CFR 54	X	X	
21 CFR 210	X		
21 CFR 211	X		
21 CFR 312	X		
21 CFR 812		X	X
21 CFR 820		X	

N/A

20 CITI

Provide the date that the members of the research team have taken the CITI training for human participants. This training must be taken within the last 4 years. Additional information can be found at: http://researchintegrity.asu.edu/training/humans

- **Gabriel Shaibi- 11/19/12**
- **Colleen Keller- 3/5/12**
- **Leopoldo Hartmann- 7/4/13**
- **Allison Williams- 8/31/12**
- **Crystal Ramos- 1/16/14**
- **Yolanda Konopken, RD, CDE- 12/18/12**
- **Micah Olson, MD – 7/17/2013**

APPROVAL: MODIFICATION

Gabriel Shaibi
CONHI - Research Faculty and Staff
602/496-0909
Gabriel.Shaibi@asu.edu

Dear Gabriel Shaibi:

On 4/8/2016 the ASU IRB reviewed the following protocol:

Type of Review:	Modification
Title:	Diabetes Prevention for Latino Youth with Prediabetes
Investigator:	Gabriel Shaibi
IRB ID:	STUDY00003735
Funding:	Name: HHS: National Institutes of Health (NIH), Grant Office ID: 2684, Funding Source ID: 1R01DK107579-01
Grant Title:	None
Grant ID:	None
Documents Reviewed:	• ELSC_Prediabetes_Prescreening_script_clean_3-22-16.pdf, Category: Recruitment Materials;<br>• ELSC Prediabetes Flyer Full page St V _04042016.pub, Category: Recruitment Materials;<br>• Completion Report-EH 12-18-12.pdf, Category: Other (to reflect anything not captured above);<br>• Shaibi - Diabetes Prevention in Latino youth with prediabetes_PROTOCOL_clean_04042016.docx, Category: IRB Protocol;<br>• Screening Phone Script_clean_03-28-16.pdf, Category: Recruitment Materials;<br>• Screening Phone Script_track changes_03-28-16.pdf, Category: Recruitment Materials;<br>• Backtranslation _Recruitmentflyer__04042016.pdf, Category: Translations;<br>• ELSC Pre-Screening Form_clean_03212016.pdf, Category: Recruitment Materials;<br>• MReason_CITI (2).pdf, Category: Other (to reflect anything not captured above);

	• Shaibi - Diabetes Prevention in Latino youth with prediabetes_PROTOCOL_track changes_04042016.docx, Category: IRB Protocol;<br>• MD Physical Activity Clearance Form.pdf, Category: Recruitment Materials;<br>• Authorization for Use and Disclosure of Protected Health Information_Spanish_03082016.pdf, Category: Other (to reflect anything not captured above);<br>• citirefresher_Ricardo_3-14-14.pdf, Category: Other (to reflect anything not captured above);<br>• Child Assent Clean, Category: Consent Form;<br>• IGT_Parental_Consent_English_trackchanges_03282016.pdf, Category: Consent Form;<br>• CITI Maria Silva 11-18-12.pdf, Category: Other (to reflect anything not captured above);<br>• MGutierrez_CITI.pdf, Category: Other (to reflect anything not captured above);<br>• PARQ.pdf, Category: Recruitment Materials;<br>• Authorization for Use and Disclosure of Protected Health Information_English_03082016.pdf, Category: Other (to reflect anything not captured above);<br>• Child Assent-MARKED, Category: Consent Form;<br>• Authorization for Use of Protected Health Information_03102016.pdf, Category: Translations;<br>• LVeleta_CITI.pdf, Category: Other (to reflect anything not captured above);<br>• Preventing Diabetes in Latino Youth - Submitted.pdf, Category: Sponsor Attachment;<br>• IGT_Parental_Consent_English_clean_03282016.pdf, Category: Consent Form;

The IRB approved the modification.

When consent is appropriate, you must use final, watermarked versions available under the “Documents” tab in ERA-IRB.

In conducting this protocol you are required to follow the requirements listed in the INVESTIGATOR MANUAL (HRP-103).

Sincerely,

IRB Administrator

cc: Allison Williams

APPROVAL: MODIFICATION

Gabriel Shaibi
CONHI - Research Faculty and Staff
602/496-0909
Gabriel.Shaibi@asu.edu

Dear Gabriel Shaibi:

On 5/11/2016 the ASU IRB reviewed the following protocol:

Type of Review:	Modification
Title:	Diabetes Prevention for Latino Youth with Prediabetes
Investigator:	Gabriel Shaibi
IRB ID:	STUDY00003735
Funding:	Name: HHS: National Institutes of Health (NIH), Grant Office ID: 2684, Funding Source ID: 1R01DK107579-01
Grant Title:	None
Grant ID:	None
Documents Reviewed:	• ELSC_Prediabetes_Prescreening_script_clean_3-22-16.pdf, Category: Recruitment Materials;<br>• ELSC Prediabetes Flyer Full page St V _04042016.pub, Category: Recruitment Materials;<br>• Hu_CITI_PCH report (Mar 2016).pdf, Category: Other (to reflect anything not captured above);<br>• Preparing for your Health Screening-Weekend_05052016.pdf, Category: Participant materials (specific directions for them);<br>• Prediabetes_Parent_Consent_Spanish_05062016.pdf, Category: Consent Form;<br>• SE and Diet.pdf, Category: Measures (Survey questions/Interview questions /interview guides/focus group questions);<br>• Prediabetes_Parental_Consent_English_clean_05032016.pdf, Category: Consent Form;<br>• Completion Report-EH 12-18-12.pdf, Category: Other (to reflect anything not captured above);<br>• Prediabetes FAQs_St.V..pdf, Category: Recruitment Materials;

	• WQOL InstrumentEnglish_Spanish.pdf, Category: Measures (Survey questions/Interview questions /interview guides/focus group questions);<br>• Prediabetes Pre-Screening Form_St.V._Spanish_04112016.pdf, Category: Recruitment Materials;<br>• ACCULTURATION AHIMSA.pdf, Category: Measures (Survey questions/Interview questions /interview guides/focus group questions);<br>• Preparing for your Testing Visit_T1-T3_Weekend_05052016.pdf, Category: Participant materials (specific directions for them);<br>• Pubertal Developmental StatusBOYS.pdf, Category: Measures (Survey questions/Interview questions /interview guides/focus group questions);<br>• Prediabetes Project Summary and Eligibility Criteria for Physicians.pdf, Category: Recruitment Materials;<br>• Preparing for your Health Screening-Weekday_05052016.pdf, Category: Participant materials (specific directions for them);<br>• SE and PA.pdf, Category: Measures (Survey questions/Interview questions /interview guides/focus group questions);<br>• Screening Phone Script_clean_03-28-16.pdf, Category: Recruitment Materials;<br>• Screening Phone Script_track changes_03-28-16.pdf, Category: Recruitment Materials;<br>• Brief Acculturation Rating Scale for Mexican Americans (ARMSA II).pdf, Category: Measures (Survey questions/Interview questions /interview guides/focus group questions);<br>• Pimentel CITI 2015.pdf, Category: Other (to reflect anything not captured above);<br>• Backtranslation _Recruitmentflyer__04042016.pdf, Category: Translations;<br>• 2007 Block Food Screener.pdf, Category: Measures (Survey questions/Interview questions /interview guides/focus group questions);<br>• SS and Diet Friends.pdf, Category: Measures (Survey questions/Interview questions /interview guides/focus group questions);<br>• Family History of Diabetes English 5-6-13.pdf, Category: Measures (Survey questions/Interview questions /interview guides/focus group questions);<br>• ELSC Pre-Screening Form_clean_03212016.pdf, Category: Recruitment Materials;<br>• MReason_CITI (2).pdf, Category: Other (to reflect anything not captured above);

	• Backtranslation _FamilyHxDiabetes_050616.pdf, Category: Translations;<br>• Demographics_youth_05052016.pdf, Category: Measures (Survey questions/Interview questions /interview guides/focus group questions);<br>• Pre-diabetes_Assent_clean_05032016.pdf, Category: Consent Form;<br>• Preparing for your Testing Visit_T1-T3_Weekday_05052016.pdf, Category: Participant materials (specific directions for them);<br>• Family History of Diabetes Spanish 5-6-13.pdf, Category: Measures (Survey questions/Interview questions /interview guides/focus group questions);<br>• Pictorial Body Image Assessment.pdf, Category: Measures (Survey questions/Interview questions /interview guides/focus group questions);<br>• MD Physical Activity Clearance Form.pdf, Category: Recruitment Materials;<br>• Lab Intake Form_05022016.pdf, Category: Measures (Survey questions/Interview questions /interview guides/focus group questions);<br>• Authorization for Use and Disclosure of Protected Health Information_Spanish_03082016.pdf, Category: Other (to reflect anything not captured above);<br>• Contact Information.pdf, Category: Measures (Survey questions/Interview questions /interview guides/focus group questions);<br>• citirefresher_Ricardo_3-14-14.pdf, Category: Other (to reflect anything not captured above);<br>• Pubertal Developmental StatusGIRLS.pdf, Category: Measures (Survey questions/Interview questions /interview guides/focus group questions);<br>• CITI Maria Silva 11-18-12.pdf, Category: Other (to reflect anything not captured above);<br>• Mexican American Cultural Values Scale.pdf, Category: Measures (Survey questions/Interview questions /interview guides/focus group questions);<br>• MGutierrez_CITI.pdf, Category: Other (to reflect anything not captured above);<br>• Shaibi - Diabetes Prevention in Latino youth with prediabetes_PROTOCOL_clean_05052016.docx, Category: IRB Protocol;<br>• PARQ.pdf, Category: Recruitment Materials;<br>• 3_Day_Physical_Activity_Recall.pdf, Category: Measures (Survey questions/Interview questions /interview guides/focus group questions);

	• Prediabetes_Parental_Consent_English_track changes_05032016.pdf, Category: Consent Form;<br>• Blood Draw_IV_Site_Care_Instructions_Bilingual_05052016_aea.pdf, Category: Participant materials (specific directions for them);<br>• Authorization for Use and Disclosure of Protected Health Information_English_03082016.pdf, Category: Other (to reflect anything not captured above);<br>• Prediabetes_ASU_Screening_Phone_Script_SPA_04192016.pdf, Category: Recruitment Materials;<br>• YQOL-SF.pdf, Category: Measures (Survey questions/Interview questions /interview guides/focus group questions);<br>• Authorization for Use of Protected Health Information_03102016.pdf, Category: Translations;<br>• Translation Certificate_05062016.pdf, Category: Translations;<br>• Pre-diabetes_Assent_trackchanges_05032016.pdf, Category: Consent Form;<br>• LVeleta_CITI.pdf, Category: Other (to reflect anything not captured above);<br>• Preventing Diabetes in Latino Youth - Submitted.pdf, Category: Sponsor Attachment;<br>• Demographics_adult.pdf, Category: Measures (Survey questions/Interview questions /interview guides/focus group questions);<br>• SS and PA Family and Friends.pdf, Category: Measures (Survey questions/Interview questions /interview guides/focus group questions);<br>• SS and Diet Family.pdf, Category: Measures (Survey questions/Interview questions /interview guides/focus group questions);<br>• Rosenberg Self-Esteem Inventory.pdf, Category: Measures (Survey questions/Interview questions /interview guides/focus group questions);<br>• Shaibi - Diabetes Prevention in Latino youth with prediabetes_PROTOCOL_trackchanges_05052016.docx, Category: IRB Protocol;

The IRB approved the modification.

When consent is appropriate, you must use final, watermarked versions available under the “Documents” tab in ERA-IRB.

In conducting this protocol you are required to follow the requirements listed in the INVESTIGATOR MANUAL (HRP-103).

Sincerely,

IRB Administrator

cc: Allison Williams
Samantha Mendez
Estela Barraza
Leopoldo Hartmann Manrique
Erica Soltero
Allison Williams
Ana Renteria Mexia
Colleen Keller
Margarita Stirk
Claudia Sanchez
Crystal Ramos
Inty Moreno
Janet McNicol
Elvia Madrid
Jessica Reyes
Janae Degroot
Felipe Castro
Stephanie Ayers

APPROVAL: MODIFICATION

Gabriel Shaibi
CONHI - Research Faculty and Staff
602/496-0909
Gabriel.Shaibi@asu.edu

Dear Gabriel Shaibi:

On 7/17/2016 the ASU IRB reviewed the following protocol:

Type of Review:	Modification
Title:	Diabetes Prevention for Latino Youth with Prediabetes
Investigator:	Gabriel Shaibi
IRB ID:	STUDY00003735
Funding:	Name: HHS: National Institutes of Health (NIH), Grant Office ID: 2684, Funding Source ID: 1R01DK107579-01
Grant Title:	None
Grant ID:	None
Documents Reviewed:	• Nutrition Label_SP.pdf, Category: Other (to reflect anything not captured above);<br>• ELSC_Prediabetes_Prescreening_script_clean_3-22-16.pdf, Category: Recruitment Materials;<br>• ELSC Prediabetes Flyer Full page St V _04042016.pub, Category: Recruitment Materials;<br>• Hu_CITI_PCH report (Mar 2016).pdf, Category: Other (to reflect anything not captured above);<br>• Preparing for your Health Screening-Weekend_05052016.pdf, Category: Participant materials (specific directions for them);<br>• SE and Diet.pdf, Category: Measures (Survey questions/Interview questions /interview guides/focus group questions);<br>• DGTipsheet34ChooseTheFoodsYouNeedToGrow.pdf, Category: Other (to reflect anything not captured above);<br>• Completion Report-EH 12-18-12.pdf, Category: Other (to reflect anything not captured above);<br>• Prediabetes FAQs_St.V..pdf, Category: Recruitment Materials;<br>•

	Prediabetes_Parental_Consent_English_trackchanges_07142016.pdf, Category: Consent Form;<br>• WQOL InstrumentEnglish_Spanish.pdf, Category: Measures (Survey questions/Interview questions /interview guides/focus group questions);<br>• Prediabetes Pre-Screening Form_St.V._Spanish_04112016.pdf, Category: Recruitment Materials;<br>• ACCULTURATION AHIMSA.pdf, Category: Measures (Survey questions/Interview questions /interview guides/focus group questions);<br>• Preparing for your Testing Visit_T1-T3_Weekend_05052016.pdf, Category: Participant materials (specific directions for them);<br>• Pubertal Developmental StatusBOYS.pdf, Category: Measures (Survey questions/Interview questions /interview guides/focus group questions);<br>• Prediabetes Project Summary and Eligibility Criteria for Physicians.pdf, Category: Recruitment Materials;<br>• Pregnancy Test Results Script_05242016.pdf, Category: Other (to reflect anything not captured above);<br>• Release of Information Sheet PCH 3 28 2013.pdf, Category: Recruitment Materials;<br>• Preparing for your Health Screening-Weekday_05052016.pdf, Category: Participant materials (specific directions for them);<br>• SE and PA.pdf, Category: Measures (Survey questions/Interview questions /interview guides/focus group questions);<br>• Screening Phone Script_clean_03-28-16.pdf, Category: Recruitment Materials;<br>• Screening Phone Script_track changes_03-28-16.pdf, Category: Recruitment Materials;<br>• DGTipsheet36EatSmartAndBeActiveAsYouGrow.pdf, Category: Other (to reflect anything not captured above);<br>• Brief Acculturation Rating Scale for Mexican Americans (ARMSA II).pdf, Category: Measures (Survey questions/Interview questions /interview guides/focus group questions);<br>• DGTipsheet34ChooseTheFoodsYouNeedToGrow-sp.pdf, Category: Other (to reflect anything not captured above);<br>• Release of Information Sheet MPHC.pdf, Category: Recruitment Materials;<br>• Nutrition Label_EG.pdf, Category: Other (to reflect anything not captured above);<br>• Pimentel CITI 2015.pdf, Category: Other (to reflect anything not captured above);<br>• Backtranslation _Recruitmentflyer__04042016.pdf, Category: Translations;<br>• 2007 Block Food Screener.pdf, Category: Measures (Survey questions/Interview questions /interview guides/focus group

	questions);<br>• SS and Diet Friends.pdf, Category: Measures (Survey questions/Interview questions /interview guides/focus group questions);<br>• Family History of Diabetes English 5-6-13.pdf, Category: Measures (Survey questions/Interview questions /interview guides/focus group questions);<br>• ELSC Pre-Screening Form_clean_03212016.pdf, Category: Recruitment Materials;<br>• DGTipsheet36EatSmartAndBeActiveAsYouGrow-sp.pdf, Category: Other (to reflect anything not captured above);<br>• MReason_CITI (2).pdf, Category: Other (to reflect anything not captured above);<br>• Pre-diabetes_Assent_clean_06302016.pdf, Category: Consent Form;<br>• Backtranslation _FamilyHxDiabetes_050616.pdf, Category: Translations;<br>• Prediabetes_Parent_Consent_Spanish_clean_07142016.pdf, Category: Consent Form;<br>• Demographics_youth_05052016.pdf, Category: Measures (Survey questions/Interview questions /interview guides/focus group questions);<br>• Preparing for your Testing Visit_T1-T3_Weekday_05052016.pdf, Category: Participant materials (specific directions for them);<br>• Family History of Diabetes Spanish 5-6-13.pdf, Category: Measures (Survey questions/Interview questions /interview guides/focus group questions);<br>• Prediabetes_Parental_Consent_English_clean_07142016.pdf, Category: Consent Form;<br>• Pictorial Body Image Assessment.pdf, Category: Measures (Survey questions/Interview questions /interview guides/focus group questions);<br>• MD-RD Notes template_07142016.pdf, Category: Other (to reflect anything not captured above);<br>• MD Physical Activity Clearance Form.pdf, Category: Recruitment Materials;<br>• Lab Intake Form_05022016.pdf, Category: Measures (Survey questions/Interview questions /interview guides/focus group questions);<br>• Authorization for Use and Disclosure of Protected Health Information_Spanish_03082016.pdf, Category: Other (to reflect anything not captured above);<br>• Contact Information.pdf, Category: Measures (Survey questions/Interview questions /interview guides/focus group questions);<br>• Pregnancy Resource List_05042016.pdf, Category: Resource list;

	• Shaibi - Diabetes Prevention in Latino youth with prediabetes_PROTOCOL_clean_07142016.docx, Category: IRB Protocol;<br>• Group 1 Notification-YMCA Directions letter_ Bilingual_06282016.pdf, Category: Participant materials (specific directions for them);<br>• citirefresher_Ricardo_3-14-14.pdf, Category: Other (to reflect anything not captured above);<br>• Group 2 Notification Letter_ Bilingual_06282016.pdf, Category: Participant materials (specific directions for them);<br>• DGTipsheet13CutBackOnSweetTreats.pdf, Category: Other (to reflect anything not captured above);<br>• Pubertal Developmental StatusGIRLS.pdf, Category: Measures (Survey questions/Interview questions /interview guides/focus group questions);<br>• Pre-diabetes_Assent_track changes_06302016.pdf, Category: Consent Form;<br>• CITI Maria Silva 11-18-12.pdf, Category: Other (to reflect anything not captured above);<br>• Mexican American Cultural Values Scale.pdf, Category: Measures (Survey questions/Interview questions /interview guides/focus group questions);<br>• MGutierrez_CITI.pdf, Category: Other (to reflect anything not captured above);<br>• Release of Information Sheet SpanishPCH.pdf, Category: Recruitment Materials;<br>• Prediabetes_Parent_Consent_Spanish_trackchanges_07142016.pdf, Category: Consent Form;<br>• PARQ.pdf, Category: Recruitment Materials;<br>• 3_Day_Physical_Activity_Recall.pdf, Category: Measures (Survey questions/Interview questions /interview guides/focus group questions);<br>• DGTipsheet13CutBackOnSweetTreats-sp.pdf, Category: Other (to reflect anything not captured above);<br>• Blood Draw_IV_Site_Care_Instructions_Bilingual_05052016_aea.pdf, Category: Participant materials (specific directions for them);<br>• Authorization for Use and Disclosure of Protected Health Information_English_03082016.pdf, Category: Other (to reflect anything not captured above);<br>• Prediabetes_ASU_Screening_Phone_Script_SPA_04192016.pdf, Category: Recruitment Materials;<br>• YQOL-SF.pdf, Category: Measures (Survey questions/Interview questions /interview guides/focus group questions);<br>• Size Up Your Servings_EG&SP.pdf, Category: Other (to reflect anything not captured above);

	• Authorization for Use of Protected Health Information_03102016.pdf, Category: Translations;<br>• PERMISSION for FUTURE CONTACT_bilingual_07112016.pdf, Category: Consent Form;<br>• Translation Certificate_05062016.pdf, Category: Translations;<br>• Backtranslation _GroupNotificationLetter_07142016.pdf, Category: Translations;<br>• LVeleta_CITI.pdf, Category: Other (to reflect anything not captured above);<br>• Preventing Diabetes in Latino Youth - Submitted.pdf, Category: Sponsor Attachment;<br>• Demographics_adult.pdf, Category: Measures (Survey questions/Interview questions /interview guides/focus group questions);<br>• SS and PA Family and Friends.pdf, Category: Measures (Survey questions/Interview questions /interview guides/focus group questions);<br>• SS and Diet Family.pdf, Category: Measures (Survey questions/Interview questions /interview guides/focus group questions);<br>• Rosenberg Self-Esteem Inventory.pdf, Category: Measures (Survey questions/Interview questions /interview guides/focus group questions);<br>• Shaibi - Diabetes Prevention in Latino youth with prediabetes_PROTOCOL_trackchanges_07142016.docx, Category: IRB Protocol;

The IRB approved the modification.

When consent is appropriate, you must use final, watermarked versions available under the “Documents” tab in ERA-IRB.

In conducting this protocol you are required to follow the requirements listed in the INVESTIGATOR MANUAL (HRP-103).

Sincerely,

IRB Administrator

cc: Allison Williams
Crystal Ramos
Elvia Madrid
Rachel Hernandez
Samantha Mendez

Allison Williams
Janae Degroot
Estela Barraza
Leopoldo Hartmann Manrique
Colleen Keller
Margarita Stirk
Stephanie Ayers
Felipe Castro
Inty Moreno
Virginia Boyd
Jessica Reyes
Ana Renteria Mexia
Erica Soltero
Claudia Sanchez
Janet McNicol
Anaid Gonzalvez

APPROVAL: MODIFICATION

Gabriel Shaibi
Health Promotion and Disease Prevention, Center for
602/496-0909
Gabriel.Shaibi@asu.edu

Dear Gabriel Shaibi:

On 12/20/2016 the ASU IRB reviewed the following protocol:

Type of Review:	Modification
Title:	Diabetes Prevention for Latino Youth with Prediabetes
Investigator:	Gabriel Shaibi
IRB ID:	STUDY00003735
Funding:	Name: HHS: National Institutes of Health (NIH), Grant Office ID: 2684, Funding Source ID: 1R01DK107579-01
Grant Title:	None
Grant ID:	None
Documents Reviewed:	• ELSC_Prediabetes_Prescreening_script_clean_3-22-16.pdf, Category: Recruitment Materials;<br>• ELSC Prediabetes Flyer Full page St V _04042016.pub, Category: Recruitment Materials;<br>• Hu_CITI_PCH report (Mar 2016).pdf, Category: Other (to reflect anything not captured above);<br>• Preparing for your Health Screening-Weekend_05052016.pdf, Category: Participant materials (specific directions for them);<br>• Shaibi - Diabetes Prevention in Latino youth with prediabetes_PROTOCOL_trackchanges_12192016.docx, Category: IRB Protocol;<br>• SE and Diet.pdf, Category: Measures (Survey questions/Interview questions /interview guides/focus group questions);<br>• Completion Report-EH 12-18-12.pdf, Category: Other (to reflect anything not captured above);<br>• PCP Engagement Letter_08-25-16.pdf, Category: Other (to reflect anything not captured above);

	• Prediabetes FAQs_St.V..pdf, Category: Recruitment Materials;<br>• WQOL InstrumentEnglish_Spanish.pdf, Category: Measures (Survey questions/Interview questions /interview guides/focus group questions);<br>• Prediabetes Pre-Screening Form_St.V._Spanish_04112016.pdf, Category: Recruitment Materials;<br>• T2-T3 Results Letter _very abnormal_10-24-16_Bilingual.pdf, Category: Participant materials (specific directions for them);<br>• ACCULTURATION AHIMSA.pdf, Category: Measures (Survey questions/Interview questions /interview guides/focus group questions);<br>• Preparing for your Testing Visit_T1-T3_Weekend_05052016.pdf, Category: Participant materials (specific directions for them);<br>• Pubertal Developmental StatusBOYS.pdf, Category: Measures (Survey questions/Interview questions /interview guides/focus group questions);<br>• Prediabetes Project Summary and Eligibility Criteria for Physicians.pdf, Category: Recruitment Materials;<br>• Notification_A1cEligibility_ PhoneScript_English_12-19-16.pdf, Category: Recruitment Materials;<br>• Preparing for your Health Screening-Weekday_05052016.pdf, Category: Participant materials (specific directions for them);<br>• SE and PA.pdf, Category: Measures (Survey questions/Interview questions /interview guides/focus group questions);<br>• Screening Phone Script_clean_03-28-16.pdf, Category: Recruitment Materials;<br>• Screening Phone Script_track changes_03-28-16.pdf, Category: Recruitment Materials;<br>• Results Letter for Ineligible_normal_10-24-16_Bilingual.pdf, Category: Participant materials (specific directions for them);<br>• Brief Acculturation Rating Scale for Mexican Americans (ARMSA II).pdf, Category: Measures (Survey questions/Interview questions /interview guides/focus group questions);<br>• Work Attendance_Parents__Spanish_11012016.pdf, Category: Measures (Survey questions/Interview questions /interview guides/focus group questions);<br>• Backtranslation _12-01-16.pdf, Category: Translations;<br>• Results Letter for Ineligible_abnormal_10-24-16_Bilingual.pdf, Category: Participant materials (specific directions for them);<br>• Pimentel CITI 2015.pdf, Category: Other (to reflect anything not captured above);<br>• Backtranslation _Recruitmentflyer__04042016.pdf, Category:

	Translations;<br>• 2007 Block Food Screener.pdf, Category: Measures (Survey questions/Interview questions /interview guides/focus group questions);<br>• SS and Diet Friends.pdf, Category: Measures (Survey questions/Interview questions /interview guides/focus group questions);<br>• Family History of Diabetes English 5-6-13.pdf, Category: Measures (Survey questions/Interview questions /interview guides/focus group questions);<br>• ELSC Pre-Screening Form_clean_03212016.pdf, Category: Recruitment Materials;<br>• MReason_CITI (2).pdf, Category: Other (to reflect anything not captured above);<br>• Backtranslation _FamilyHxDiabetes_050616.pdf, Category: Translations;<br>• Demographics_youth_05052016.pdf, Category: Measures (Survey questions/Interview questions /interview guides/focus group questions);<br>• Preparing for your Testing Visit_T1-T3_Weekday_05052016.pdf, Category: Participant materials (specific directions for them);<br>• Family History of Diabetes Spanish 5-6-13.pdf, Category: Measures (Survey questions/Interview questions /interview guides/focus group questions);<br>• PCP letter-lab results_11-10-16.pdf, Category: Other (to reflect anything not captured above);<br>• Shaibi - Diabetes Prevention in Latino youth with prediabetes_PROTOCOL_clean_12192016.docx, Category: IRB Protocol;<br>• Work Attendance_Youth_10-27-16.pdf, Category: Measures (Survey questions/Interview questions /interview guides/focus group questions);<br>• T2-T3 Results Letter_abnormal_10-24-16_Bilingual.pdf, Category: Participant materials (specific directions for them);<br>• Pictorial Body Image Assessment.pdf, Category: Measures (Survey questions/Interview questions /interview guides/focus group questions);<br>• Backtranslation _11-10-16.pdf, Category: Translations;<br>• MD Physical Activity Clearance Form.pdf, Category: Recruitment Materials;<br>• Lab Intake Form_05022016.pdf, Category: Measures (Survey questions/Interview questions /interview guides/focus group questions);<br>• Authorization for Use and Disclosure of Protected Health Information_Spanish_03082016.pdf, Category: Other (to reflect

	anything not captured above);<br>• Contact Information.pdf, Category: Measures (Survey questions/Interview questions /interview guides/focus group questions);<br>• Results Letter for Ineligible_very abnormal_10-24-16_Bilingual.pdf, Category: Participant materials (specific directions for them);<br>• citirefresher_Ricardo_3-14-14.pdf, Category: Other (to reflect anything not captured above);<br>• Pubertal Developmental StatusGIRLS.pdf, Category: Measures (Survey questions/Interview questions /interview guides/focus group questions);<br>• CITI Maria Silva 11-18-12.pdf, Category: Other (to reflect anything not captured above);<br>• Mexican American Cultural Values Scale.pdf, Category: Measures (Survey questions/Interview questions /interview guides/focus group questions);<br>• Pre-diabetes_Assent_clean_09292016.pdf, Category: Consent Form;<br>• PCP Recruitment Letter to Patients_Bilingual_1212016.pdf, Category: Recruitment Materials;<br>• MGutierrez_CITI.pdf, Category: Other (to reflect anything not captured above);<br>• Work Attendance_Parents_10-27-16.pdf, Category: Measures (Survey questions/Interview questions /interview guides/focus group questions);<br>• PARQ.pdf, Category: Recruitment Materials;<br>• 3_Day_Physical_Activity_Recall.pdf, Category: Measures (Survey questions/Interview questions /interview guides/focus group questions);<br>• Blood Draw_IV_Site_Care_Instructions_Bilingual_05052016_aea.pdf, Category: Participant materials (specific directions for them);<br>• T2-T3 Results Letter _normal_10-24-16_Bilingual.pdf, Category: Participant materials (specific directions for them);<br>• Prediabetes_Parent_Consent_Spanish_clean_11-10-2016.pdf, Category: Consent Form;<br>• Authorization for Use and Disclosure of Protected Health Information_English_03082016.pdf, Category: Other (to reflect anything not captured above);<br>• Prediabetes_ASU_Screening_Phone_Script_SPA_04192016.pdf, Category: Recruitment Materials;<br>• YQOL-SF.pdf, Category: Measures (Survey questions/Interview questions /interview guides/focus group questions);

	• Authorization for Use of Protected Health Information_03102016.pdf, Category: Translations;<br>• Translation Certificate_05062016.pdf, Category: Translations;<br>• LVeleta_CITI.pdf, Category: Other (to reflect anything not captured above);<br>• Prediabetes_Parental_Consent_English_clean_11-10-2016.pdf, Category: Consent Form;<br>• Preventing Diabetes in Latino Youth - Submitted.pdf, Category: Sponsor Attachment;<br>• Demographics_adult.pdf, Category: Measures (Survey questions/Interview questions /interview guides/focus group questions);<br>• SS and PA Family and Friends.pdf, Category: Measures (Survey questions/Interview questions /interview guides/focus group questions);<br>• SS and Diet Family.pdf, Category: Measures (Survey questions/Interview questions /interview guides/focus group questions);<br>• Rosenberg Self-Esteem Inventory.pdf, Category: Measures (Survey questions/Interview questions /interview guides/focus group questions);<br>• FAQs for Providers_08252016.pdf, Category: Other (to reflect anything not captured above);

The IRB approved the modification.

When consent is appropriate, you must use final, watermarked versions available under the "Documents" tab in ERA-IRB.

In conducting this protocol you are required to follow the requirements listed in the INVESTIGATOR MANUAL (HRP-103).

Sincerely,

IRB Administrator

cc: Allison Williams
Allison Williams
Samantha Mendez
Margarita Stirk
Ana Renteria Mexia
Janae Degroot
Crystal Ramos
Rachel Hernandez

Zari Chacon
Arlene Ramos
Virginia Boyd
Anaid Gonzalvez
Inty Moreno
Felipe Castro
Stephanie Ayers
Jessica Reyes
Colleen Keller
Claudia Sanchez
Janet McNicol
Leopoldo Hartmann Manrique
Estela Barraza
Erica Soltero
Elvia Madrid

ASU Knowledge Enterprise Development

APPROVAL: MODIFICATION

Gabriel Shaibi
Health Promotion and Disease Prevention, Center for
602/496-0909
Gabriel.Shaibi@asu.edu

Dear Gabriel Shaibi:

On 2/19/2017 the ASU IRB reviewed the following protocol:

Type of Review:	Modification
Title:	Diabetes Prevention for Latino Youth with Prediabetes
Investigator:	Gabriel Shaibi
IRB ID:	STUDY00003735
Funding:	Name: HHS: National Institutes of Health (NIH), Grant Office ID: 2684, Funding Source ID: 1R01DK107579-01
Grant Title:	None
Grant ID:	None
Documents Reviewed:	• ELSC_Prediabetes_Prescreening_script_clean_3-22-16.pdf, Category: Recruitment Materials;<br>• ELSC Prediabetes Flyer Full page St V _04042016.pub, Category: Recruitment Materials;<br>• Hu_CITI_PCH report (Mar 2016).pdf, Category: Other (to reflect anything not captured above);<br>• Preparing for your Health Screening-Weekend_05052016.pdf, Category: Participant materials (specific directions for them);<br>• SE and Diet.pdf, Category: Measures (Survey questions/Interview questions /interview guides/focus group questions);<br>• Completion Report-EH 12-18-12.pdf, Category: Other (to reflect anything not captured above);<br>• PCP Engagement Letter_08-25-16.pdf, Category: Other (to reflect anything not captured above);<br>• Prediabetes FAQs_St.V..pdf, Category: Recruitment Materials;<br>• WQOL InstrumentEnglish_Spanish.pdf, Category: Measures (Survey questions/Interview questions /interview guides/focus group questions);

	• Prediabetes Pre-Screening Form_St.V._Spanish_04112016.pdf, Category: Recruitment Materials;<br>• T2-T3 Results Letter _very abnormal_10-24-16_Bilingual.pdf, Category: Participant materials (specific directions for them);<br>• ACCULTURATION AHIMSA.pdf, Category: Measures (Survey questions/Interview questions /interview guides/focus group questions);<br>• Preparing for your Testing Visit_T1-T3_Weekend_05052016.pdf, Category: Participant materials (specific directions for them);<br>• Pubertal Developmental StatusBOYS.pdf, Category: Measures (Survey questions/Interview questions /interview guides/focus group questions);<br>• Prediabetes Project Summary and Eligibility Criteria for Physicians.pdf, Category: Recruitment Materials;<br>• Notification_A1cEligibility_ PhoneScript_English_12-19-16.pdf, Category: Recruitment Materials;<br>• Preparing for your Health Screening-Weekday_05052016.pdf, Category: Participant materials (specific directions for them);<br>• SE and PA.pdf, Category: Measures (Survey questions/Interview questions /interview guides/focus group questions);<br>• Screening Phone Script_clean_03-28-16.pdf, Category: Recruitment Materials;<br>• Screening Phone Script_track changes_03-28-16.pdf, Category: Recruitment Materials;<br>• Results Letter for Ineligible_normal_10-24-16_Bilingual.pdf, Category: Participant materials (specific directions for them);<br>• Shaibi - Diabetes Prevention in Latino youth with prediabetes_PROTOCOL_track changes_02132017.docx, Category: IRB Protocol;<br>• Brief Acculturation Rating Scale for Mexican Americans (ARMSA II).pdf, Category: Measures (Survey questions/Interview questions /interview guides/focus group questions);<br>• Work Attendance_Parents__Spanish_11012016.pdf, Category: Measures (Survey questions/Interview questions /interview guides/focus group questions);<br>• Backtranslation _12-01-16.pdf, Category: Translations;<br>• Results Letter for Ineligible_abnormal_10-24-16_Bilingual.pdf, Category: Participant materials (specific directions for them);<br>• Pimentel CITI 2015.pdf, Category: Other (to reflect anything not captured above);<br>• Backtranslation _Recruitmentflyer__04042016.pdf, Category: Translations;<br>• 2007 Block Food Screener.pdf, Category: Measures (Survey questions/Interview questions /interview guides/focus group questions);<br>• SS and Diet Friends.pdf, Category: Measures (Survey

	questions/Interview questions /interview guides/focus group questions);<br>• Pre-diabetes_Assent_trackchanges_02132017.pdf, Category: Consent Form;<br>• Family History of Diabetes English 5-6-13.pdf, Category: Measures (Survey questions/Interview questions /interview guides/focus group questions);<br>• ELSC Pre-Screening Form_clean_03212016.pdf, Category: Recruitment Materials;<br>• MReason_CITI (2).pdf, Category: Other (to reflect anything not captured above);<br>• Backtranslation _FamilyHxDiabetes_050616.pdf, Category: Translations;<br>• Demographics_youth_05052016.pdf, Category: Measures (Survey questions/Interview questions /interview guides/focus group questions);<br>• Preparing for your Testing Visit_T1-T3_Weekday_05052016.pdf, Category: Participant materials (specific directions for them);<br>• Family History of Diabetes Spanish 5-6-13.pdf, Category: Measures (Survey questions/Interview questions /interview guides/focus group questions);<br>• PCP letter-lab results_11-10-16.pdf, Category: Other (to reflect anything not captured above);<br>• Work Attendance_Youth_10-27-16.pdf, Category: Measures (Survey questions/Interview questions /interview guides/focus group questions);<br>• T2-T3 Results Letter_abnormal_10-24-16_Bilingual.pdf, Category: Participant materials (specific directions for them);<br>• Pictorial Body Image Assessment.pdf, Category: Measures (Survey questions/Interview questions /interview guides/focus group questions);<br>• Backtranslation _11-10-16.pdf, Category: Translations;<br>• MD Physical Activity Clearance Form.pdf, Category: Recruitment Materials;<br>• Lab Intake Form_05022016.pdf, Category: Measures (Survey questions/Interview questions /interview guides/focus group questions);<br>• Shaibi - Diabetes Prevention in Latino youth with prediabetes_PROTOCOL_clean_02132017.docx, Category: IRB Protocol;<br>• Authorization for Use and Disclosure of Protected Health Information_Spanish_03082016.pdf, Category: Other (to reflect anything not captured above);<br>• Contact Information.pdf, Category: Measures (Survey questions/Interview questions /interview guides/focus group questions);

	• Results Letter for Ineligible_very abnormal_10-24-16_Bilingual.pdf, Category: Participant materials (specific directions for them);<br>• Prediabetes_Parent_Consent_Spanish_track changes_02132017.pdf, Category: Consent Form;<br>• citirefresher_Ricardo_3-14-14.pdf, Category: Other (to reflect anything not captured above);<br>• Pubertal Developmental StatusGIRLS.pdf, Category: Measures (Survey questions/Interview questions /interview guides/focus group questions);<br>• CITI Maria Silva 11-18-12.pdf, Category: Other (to reflect anything not captured above);<br>• Mexican American Cultural Values Scale.pdf, Category: Measures (Survey questions/Interview questions /interview guides/focus group questions);<br>• Backtranslation _2-13-17.pdf, Category: Translations;<br>• PCP Recruitment Letter to Patients_Bilingual_1212016.pdf, Category: Recruitment Materials;<br>• MGutierrez_CITI.pdf, Category: Other (to reflect anything not captured above);<br>• Work Attendance_Parents_10-27-16.pdf, Category: Measures (Survey questions/Interview questions /interview guides/focus group questions);<br>• Pre-diabetes_Assent_clean_0213207.pdf, Category: Consent Form;<br>• PARQ.pdf, Category: Recruitment Materials;<br>• 3_Day_Physical_Activity_Recall.pdf, Category: Measures (Survey questions/Interview questions /interview guides/focus group questions);<br>• Prediabetes_Parent_Consent_Spanish_clean_02132017.pdf, Category: Consent Form;<br>• Prediabetes_Parental_Consent_English_trackchanges_02132017.pdf, Category: Consent Form;<br>• Blood Draw_IV_Site_Care_Instructions_Bilingual_05052016_aea.pdf, Category: Participant materials (specific directions for them);<br>• T2-T3 Results Letter _normal_10-24-16_Bilingual.pdf, Category: Participant materials (specific directions for them);<br>• Authorization for Use and Disclosure of Protected Health Information_English_03082016.pdf, Category: Other (to reflect anything not captured above);<br>• Prediabetes_ASU_Screening_Phone_Script_SPA_04192016.pdf, Category: Recruitment Materials;<br>• YQOL-SF.pdf, Category: Measures (Survey questions/Interview questions /interview guides/focus group questions);<br>• Authorization for Use of Protected Health

	Information_03102016.pdf, Category: Translations;<br>• Translation Certificate_05062016.pdf, Category: Translations;<br>• LVeleta_CITI.pdf, Category: Other (to reflect anything not captured above);<br>• Preventing Diabetes in Latino Youth - Submitted.pdf, Category: Sponsor Attachment;<br>• Demographics_adult.pdf, Category: Measures (Survey questions/Interview questions /interview guides/focus group questions);<br>• SS and PA Family and Friends.pdf, Category: Measures (Survey questions/Interview questions /interview guides/focus group questions);<br>• SS and Diet Family.pdf, Category: Measures (Survey questions/Interview questions /interview guides/focus group questions);<br>• Prediabetes_Parental_Consent_English_clean_02132017.pdf, Category: Consent Form;<br>• Rosenberg Self-Esteem Inventory.pdf, Category: Measures (Survey questions/Interview questions /interview guides/focus group questions);<br>• FAQs for Providers_08252016.pdf, Category: Other (to reflect anything not captured above);

The IRB approved the modification.

When consent is appropriate, you must use final, watermarked versions available under the "Documents" tab in ERA-IRB.

In conducting this protocol you are required to follow the requirements listed in the INVESTIGATOR MANUAL (HRP-103).

Sincerely,

IRB Administrator

cc: Allison Williams
Allison Williams
Samantha Mendez
Margarita Stirk
Ana Renteria Mexia
Janae Degroot
Crystal Ramos
Rachel Hernandez
Zari Chacon

Arlene Ramos
Virginia Boyd
Anaid Gonzalvez
Inty Moreno
Felipe Castro
Stephanie Ayers
Jessica Reyes
Colleen Keller
Claudia Sanchez
Janet McNicol
Leopoldo Hartmann Manrique
Estela Barraza
Erica Soltero
Elvia Madrid

APPROVAL: MODIFICATION

Gabriel Shaibi
Health Promotion and Disease Prevention, Center for
602/496-0909
Gabriel.Shaibi@asu.edu

Dear Gabriel Shaibi:

On 2/23/2017 the ASU IRB reviewed the following protocol:

Type of Review:	Modification
Title:	Diabetes Prevention for Latino Youth with Prediabetes
Investigator:	Gabriel Shaibi
IRB ID:	STUDY00003735
Funding:	Name: HHS: National Institutes of Health (NIH), Grant Office ID: 2684, Funding Source ID: 1R01DK107579-01
Grant Title:	None
Grant ID:	None
Documents Reviewed:	• ELSC_Prediabetes_Prescreening_script_clean_3-22-16.pdf, Category: Recruitment Materials; • ELSC Prediabetes Flyer Full page St V _04042016.pub, Category: Recruitment Materials; • Hu_CITI_PCH report (Mar 2016).pdf, Category: Other (to reflect anything not captured above); • Preparing for your Health Screening-Weekend_05052016.pdf, Category: Participant materials (specific directions for them); • SE and Diet.pdf, Category: Measures (Survey questions/Interview questions /interview guides/focus group questions); • Completion Report-EH 12-18-12.pdf, Category: Other (to reflect anything not captured above); • PCP Engagement Letter_08-25-16.pdf, Category: Other (to reflect anything not captured above); • Prediabetes FAQs_St.V..pdf, Category: Recruitment Materials; • WQOL InstrumentEnglish_Spanish.pdf, Category: Measures (Survey questions/Interview questions /interview guides/focus group questions);

	• Prediabetes Pre-Screening Form_St.V._Spanish_04112016.pdf, Category: Recruitment Materials;<br>• T2-T3 Results Letter _very abnormal_10-24-16_Bilingual.pdf, Category: Participant materials (specific directions for them);<br>• ACCULTURATION AHIMSA.pdf, Category: Measures (Survey questions/Interview questions /interview guides/focus group questions);<br>• Preparing for your Testing Visit_T1-T3_Weekend_05052016.pdf, Category: Participant materials (specific directions for them);<br>• Pubertal Developmental StatusBOYS.pdf, Category: Measures (Survey questions/Interview questions /interview guides/focus group questions);<br>• Prediabetes Project Summary and Eligibility Criteria for Physicians.pdf, Category: Recruitment Materials;<br>• Shaibi - Diabetes Prevention in Latino youth with prediabetes_PROTOCOL_track changes_02232017.docx, Category: IRB Protocol;<br>• Notification_A1cEligibility_ PhoneScript_English_12-19-16.pdf, Category: Recruitment Materials;<br>• Preparing for your Health Screening-Weekday_05052016.pdf, Category: Participant materials (specific directions for them);<br>• SE and PA.pdf, Category: Measures (Survey questions/Interview questions /interview guides/focus group questions);<br>• Screening Phone Script_clean_03-28-16.pdf, Category: Recruitment Materials;<br>• Screening Phone Script_track changes_03-28-16.pdf, Category: Recruitment Materials;<br>• Results Letter for Ineligible_normal_10-24-16_Bilingual.pdf, Category: Participant materials (specific directions for them);<br>• Brief Acculturation Rating Scale for Mexican Americans (ARMSA II).pdf, Category: Measures (Survey questions/Interview questions /interview guides/focus group questions);<br>• Work Attendance_Parents__Spanish_11012016.pdf, Category: Measures (Survey questions/Interview questions /interview guides/focus group questions);<br>• Backtranslation _12-01-16.pdf, Category: Translations;<br>• Results Letter for Ineligible_abnormal_10-24-16_Bilingual.pdf, Category: Participant materials (specific directions for them);<br>• Pimentel CITI 2015.pdf, Category: Other (to reflect anything not captured above);<br>• Backtranslation _Recruitmentflyer__04042016.pdf, Category: Translations;<br>• 2007 Block Food Screener.pdf, Category: Measures (Survey questions/Interview questions /interview guides/focus group questions);<br>• SS and Diet Friends.pdf, Category: Measures (Survey

	questions/Interview questions /interview guides/focus group questions);<br>• Pre-diabetes_Assent_trackchanges_02132017.pdf, Category: Consent Form;<br>• Family History of Diabetes English 5-6-13.pdf, Category: Measures (Survey questions/Interview questions /interview guides/focus group questions);<br>• ELSC Pre-Screening Form_clean_03212016.pdf, Category: Recruitment Materials;<br>• Shaibi - Diabetes Prevention in Latino youth with prediabetes_PROTOCOL_clean_02232017.docx, Category: IRB Protocol;<br>• MReason_CITI (2).pdf, Category: Other (to reflect anything not captured above);<br>• Backtranslation _FamilyHxDiabetes_050616.pdf, Category: Translations;<br>• MD-Provider Clearance Letter_SSRI.pdf, Category: Recruitment Materials;<br>• Demographics_youth_05052016.pdf, Category: Measures (Survey questions/Interview questions /interview guides/focus group questions);<br>• Preparing for your Testing Visit_T1-T3_Weekday_05052016.pdf, Category: Participant materials (specific directions for them);<br>• Family History of Diabetes Spanish 5-6-13.pdf, Category: Measures (Survey questions/Interview questions /interview guides/focus group questions);<br>• PCP letter-lab results_11-10-16.pdf, Category: Other (to reflect anything not captured above);<br>• Work Attendance_Youth_10-27-16.pdf, Category: Measures (Survey questions/Interview questions /interview guides/focus group questions);<br>• T2-T3 Results Letter_abnormal_10-24-16_Bilingual.pdf, Category: Participant materials (specific directions for them);<br>• Pictorial Body Image Assessment.pdf, Category: Measures (Survey questions/Interview questions /interview guides/focus group questions);<br>• Backtranslation _11-10-16.pdf, Category: Translations;<br>• MD Physical Activity Clearance Form.pdf, Category: Recruitment Materials;<br>• Lab Intake Form_05022016.pdf, Category: Measures (Survey questions/Interview questions /interview guides/focus group questions);<br>• Authorization for Use and Disclosure of Protected Health Information_Spanish_03082016.pdf, Category: Other (to reflect anything not captured above);<br>• Contact Information.pdf, Category: Measures (Survey

	questions/Interview questions /interview guides/focus group questions);<br>• Results Letter for Ineligible_very abnormal_10-24-16_Bilingual.pdf, Category: Participant materials (specific directions for them);<br>• Prediabetes_Parent_Consent_Spanish_track changes_02132017.pdf, Category: Consent Form;<br>• citirefresher_Ricardo_3-14-14.pdf, Category: Other (to reflect anything not captured above);<br>• Pubertal Developmental StatusGIRLS.pdf, Category: Measures (Survey questions/Interview questions /interview guides/focus group questions);<br>• CITI Maria Silva 11-18-12.pdf, Category: Other (to reflect anything not captured above);<br>• Mexican American Cultural Values Scale.pdf, Category: Measures (Survey questions/Interview questions /interview guides/focus group questions);<br>• Backtranslation _2-13-17.pdf, Category: Translations;<br>• PCP Recruitment Letter to Patients_Bilingual_1212016.pdf, Category: Recruitment Materials;<br>• MGutierrez_CITI.pdf, Category: Other (to reflect anything not captured above);<br>• Work Attendance_Parents_10-27-16.pdf, Category: Measures (Survey questions/Interview questions /interview guides/focus group questions);<br>• Pre-diabetes_Assent_clean_0213207.pdf, Category: Consent Form;<br>• PARQ.pdf, Category: Recruitment Materials;<br>• 3_Day_Physical_Activity_Recall.pdf, Category: Measures (Survey questions/Interview questions /interview guides/focus group questions);<br>• Prediabetes_Parent_Consent_Spanish_clean_02132017.pdf, Category: Consent Form;<br>• Prediabetes_Parental_Consent_English_trackchanges_02132017.pdf, Category: Consent Form;<br>• Blood Draw_IV_Site_Care_Instructions_Bilingual_05052016_aea.pdf, Category: Participant materials (specific directions for them);<br>• T2-T3 Results Letter _normal_10-24-16_Bilingual.pdf, Category: Participant materials (specific directions for them);<br>• Authorization for Use and Disclosure of Protected Health Information_English_03082016.pdf, Category: Other (to reflect anything not captured above);<br>• Prediabetes_ASU_Screening_Phone_Script_SPA_04192016.pdf, Category: Recruitment Materials;<br>• YQOL-SF.pdf, Category: Measures (Survey questions/Interview

	questions /interview guides/focus group questions);<br>• Authorization for Use of Protected Health Information_03102016.pdf, Category: Translations;<br>• Translation Certificate_05062016.pdf, Category: Translations;<br>• LVeleta_CITI.pdf, Category: Other (to reflect anything not captured above);<br>• Preventing Diabetes in Latino Youth - Submitted.pdf, Category: Sponsor Attachment;<br>• Demographics_adult.pdf, Category: Measures (Survey questions/Interview questions /interview guides/focus group questions);<br>• SS and PA Family and Friends.pdf, Category: Measures (Survey questions/Interview questions /interview guides/focus group questions);<br>• SS and Diet Family.pdf, Category: Measures (Survey questions/Interview questions /interview guides/focus group questions);<br>• Prediabetes_Parental_Consent_English_clean_02132017.pdf, Category: Consent Form;<br>• Rosenberg Self-Esteem Inventory.pdf, Category: Measures (Survey questions/Interview questions /interview guides/focus group questions);<br>• FAQs for Providers_08252016.pdf, Category: Other (to reflect anything not captured above);

The IRB approved the modification.

When consent is appropriate, you must use final, watermarked versions available under the “Documents” tab in ERA-IRB.

In conducting this protocol you are required to follow the requirements listed in the INVESTIGATOR MANUAL (HRP-103).

Sincerely,

IRB Administrator

cc: Allison Williams
Crystal Ramos
Allison Williams
Virginia Boyd
Rachel Hernandez
Tatianna Alvarado
Erica Soltero

Inty Moreno
Stephanie Ayers
Samantha Mendez
Janet McNicol
Jessica Reyes
Janae Degroot
Anaid Gonzalvez
Leopoldo Hartmann Manrique
Felipe Castro
Margarita Stirk
Elvia Madrid
Arlene Ramos
Ana Renteria Mexia
Zari Chacon
Colleen Keller
Claudia Sanchez
Estela Barraza

APPROVAL: MODIFICATION

Gabriel Shaibi
Health Promotion and Disease Prevention, Center for
602/496-0909
Gabriel.Shaibi@asu.edu

Dear Gabriel Shaibi:

On 3/7/2017 the ASU IRB reviewed the following protocol:

Type of Review:	Modification
Title:	Diabetes Prevention for Latino Youth with Prediabetes
Investigator:	Gabriel Shaibi
IRB ID:	STUDY00003735
Funding:	Name: HHS: National Institutes of Health (NIH), Grant Office ID: 2684, Funding Source ID: 1R01DK107579-01
Grant Title:	None
Grant ID:	None
Documents Reviewed:	• ELSC_Prediabetes_Prescreening_script_clean_3-22-16.pdf, Category: Recruitment Materials;<br>• ELSC Prediabetes Flyer Full page St V _04042016.pub, Category: Recruitment Materials;<br>• Hu_CITI_PCH report (Mar 2016).pdf, Category: Other (to reflect anything not captured above);<br>• Preparing for your Health Screening-Weekend_05052016.pdf, Category: Participant materials (specific directions for them);<br>• SE and Diet.pdf, Category: Measures (Survey questions/Interview questions /interview guides/focus group questions);<br>• Completion Report-EH 12-18-12.pdf, Category: Other (to reflect anything not captured above);<br>• PCP Engagement Letter_08-25-16.pdf, Category: Other (to reflect anything not captured above);<br>• Prediabetes FAQs_St.V..pdf, Category: Recruitment Materials;<br>• WQOL InstrumentEnglish_Spanish.pdf, Category: Measures (Survey questions/Interview questions /interview guides/focus group questions);

	• Prediabetes Pre-Screening Form_St.V._Spanish_04112016.pdf, Category: Recruitment Materials;<br>• T2-T3 Results Letter _very abnormal_10-24-16_Bilingual.pdf, Category: Participant materials (specific directions for them);<br>• ACCULTURATION AHIMSA.pdf, Category: Measures (Survey questions/Interview questions /interview guides/focus group questions);<br>• Preparing for your Testing Visit_T1-T3_Weekend_05052016.pdf, Category: Participant materials (specific directions for them);<br>• Pubertal Developmental StatusBOYS.pdf, Category: Measures (Survey questions/Interview questions /interview guides/focus group questions);<br>• Prediabetes Project Summary and Eligibility Criteria for Physicians.pdf, Category: Recruitment Materials;<br>• Shaibi - Diabetes Prevention in Latino youth with prediabetes_PROTOCOL_track changes_02232017.docx, Category: IRB Protocol;<br>• Notification_A1cEligibility_ PhoneScript_English_12-19-16.pdf, Category: Recruitment Materials;<br>• Preparing for your Health Screening-Weekday_05052016.pdf, Category: Participant materials (specific directions for them);<br>• SE and PA.pdf, Category: Measures (Survey questions/Interview questions /interview guides/focus group questions);<br>• Screening Phone Script_clean_03-28-16.pdf, Category: Recruitment Materials;<br>• Screening Phone Script_track changes_03-28-16.pdf, Category: Recruitment Materials;<br>• Results Letter for Ineligible_normal_10-24-16_Bilingual.pdf, Category: Participant materials (specific directions for them);<br>• Brief Acculturation Rating Scale for Mexican Americans (ARMSA II).pdf, Category: Measures (Survey questions/Interview questions /interview guides/focus group questions);<br>• Work Attendance_Parents__Spanish_11012016.pdf, Category: Measures (Survey questions/Interview questions /interview guides/focus group questions);<br>• Backtranslation _12-01-16.pdf, Category: Translations;<br>• Results Letter for Ineligible_abnormal_10-24-16_Bilingual.pdf, Category: Participant materials (specific directions for them);<br>• Pimentel CITI 2015.pdf, Category: Other (to reflect anything not captured above);<br>• Tachicardia_parent_Letter_Spanish_03062017.pdf, Category: Participant materials (specific directions for them);<br>• Backtranslation _Recruitmentflyer__04042016.pdf, Category: Translations;<br>• 2007 Block Food Screener.pdf, Category: Measures (Survey questions/Interview questions /interview guides/focus group

	questions);<br>• SS and Diet Friends.pdf, Category: Measures (Survey questions/Interview questions /interview guides/focus group questions);<br>• Pre-diabetes_Assent_trackchanges_02132017.pdf, Category: Consent Form;<br>• Family History of Diabetes English 5-6-13.pdf, Category: Measures (Survey questions/Interview questions /interview guides/focus group questions);<br>• ELSC Pre-Screening Form_clean_03212016.pdf, Category: Recruitment Materials;<br>• Shaibi - Diabetes Prevention in Latino youth with prediabetes_PROTOCOL_clean_02232017.docx, Category: IRB Protocol;<br>• MReason_CITI (2).pdf, Category: Other (to reflect anything not captured above);<br>• Backtranslation _FamilyHxDiabetes_050616.pdf, Category: Translations;<br>• MD-Provider Clearance Letter_SSRI.pdf, Category: Recruitment Materials;<br>• Tachycardia_parent_letter_02272017.pdf, Category: Participant materials (specific directions for them);<br>• Backtranslation_03062017.pdf, Category: Translations;<br>• Demographics_youth_05052016.pdf, Category: Measures (Survey questions/Interview questions /interview guides/focus group questions);<br>• Preparing for your Testing Visit_T1-T3_Weekday_05052016.pdf, Category: Participant materials (specific directions for them);<br>• SOP tachycardia.pdf, Category: Other (to reflect anything not captured above);<br>• Family History of Diabetes Spanish 5-6-13.pdf, Category: Measures (Survey questions/Interview questions /interview guides/focus group questions);<br>• PCP letter-lab results_11-10-16.pdf, Category: Other (to reflect anything not captured above);<br>• Work Attendance_Youth_10-27-16.pdf, Category: Measures (Survey questions/Interview questions /interview guides/focus group questions);<br>• T2-T3 Results Letter_abnormal_10-24-16_Bilingual.pdf, Category: Participant materials (specific directions for them);<br>• Pictorial Body Image Assessment.pdf, Category: Measures (Survey questions/Interview questions /interview guides/focus group questions);<br>• Backtranslation _11-10-16.pdf, Category: Translations;<br>• MD Physical Activity Clearance Form.pdf, Category: Recruitment Materials;

	• Lab Intake Form_05022016.pdf, Category: Measures (Survey questions/Interview questions /interview guides/focus group questions);<br>• Authorization for Use and Disclosure of Protected Health Information_Spanish_03082016.pdf, Category: Other (to reflect anything not captured above);<br>• Contact Information.pdf, Category: Measures (Survey questions/Interview questions /interview guides/focus group questions);<br>• Results Letter for Ineligible_very abnormal_10-24-16_Bilingual.pdf, Category: Participant materials (specific directions for them);<br>• Prediabetes_Parent_Consent_Spanish_track changes_02132017.pdf, Category: Consent Form;<br>• citirefresher_Ricardo_3-14-14.pdf, Category: Other (to reflect anything not captured above);<br>• Pubertal Developmental StatusGIRLS.pdf, Category: Measures (Survey questions/Interview questions /interview guides/focus group questions);<br>• CITI Maria Silva 11-18-12.pdf, Category: Other (to reflect anything not captured above);<br>• Mexican American Cultural Values Scale.pdf, Category: Measures (Survey questions/Interview questions /interview guides/focus group questions);<br>• Backtranslation _2-13-17.pdf, Category: Translations;<br>• PCP Recruitment Letter to Patients_Bilingual_1212016.pdf, Category: Recruitment Materials;<br>• MGutierrez_CITI.pdf, Category: Other (to reflect anything not captured above);<br>• Work Attendance_Parents_10-27-16.pdf, Category: Measures (Survey questions/Interview questions /interview guides/focus group questions);<br>• Pre-diabetes_Assent_clean_0213207.pdf, Category: Consent Form;<br>• PARQ.pdf, Category: Recruitment Materials;<br>• 3_Day_Physical_Activity_Recall.pdf, Category: Measures (Survey questions/Interview questions /interview guides/focus group questions);<br>• Prediabetes_Parent_Consent_Spanish_clean_02132017.pdf, Category: Consent Form;<br>• Prediabetes_Parental_Consent_English_trackchanges_02132017.pdf, Category: Consent Form;<br>• Blood Draw_IV_Site_Care_Instructions_Bilingual_05052016_aea.pdf, Category: Participant materials (specific directions for them);<br>• T2-T3 Results Letter _normal_10-24-16_Bilingual.pdf, Category:

	Participant materials (specific directions for them);<br>• Authorization for Use and Disclosure of Protected Health Information_English_03082016.pdf, Category: Other (to reflect anything not captured above);<br>• Prediabetes_ASU_Screening_Phone_Script_SPA_04192016.pdf, Category: Recruitment Materials;<br>• YQOL-SF.pdf, Category: Measures (Survey questions/Interview questions /interview guides/focus group questions);<br>• Authorization for Use of Protected Health Information_03102016.pdf, Category: Translations;<br>• Translation Certificate_05062016.pdf, Category: Translations;<br>• LVeleta_CITI.pdf, Category: Other (to reflect anything not captured above);<br>• Preventing Diabetes in Latino Youth - Submitted.pdf, Category: Sponsor Attachment;<br>• Demographics_adult.pdf, Category: Measures (Survey questions/Interview questions /interview guides/focus group questions);<br>• SS and PA Family and Friends.pdf, Category: Measures (Survey questions/Interview questions /interview guides/focus group questions);<br>• SS and Diet Family.pdf, Category: Measures (Survey questions/Interview questions /interview guides/focus group questions);<br>• Prediabetes_Parental_Consent_English_clean_02132017.pdf, Category: Consent Form;<br>• Rosenberg Self-Esteem Inventory.pdf, Category: Measures (Survey questions/Interview questions /interview guides/focus group questions);<br>• FAQs for Providers_08252016.pdf, Category: Other (to reflect anything not captured above);

The IRB approved the modification.

When consent is appropriate, you must use final, watermarked versions available under the “Documents” tab in ERA-IRB.

In conducting this protocol you are required to follow the requirements listed in the INVESTIGATOR MANUAL (HRP-103).

Sincerely,

IRB Administrator

cc: Allison Williams
Crystal Ramos
Allison Williams
Virginia Boyd
Rachel Hernandez
Tatianna Alvarado
Erica Soltero
Inty Moreno
Stephanie Ayers
Samantha Mendez
Janet McNicol
Jessica Reyes
Janae Degroot
Anaid Gonzalvez
Leopoldo Hartmann Manrique
Felipe Castro
Margarita Stirk
Elvia Madrid
Arlene Ramos
Ana Renteria Mexia
Zari Chacon
Colleen Keller
Claudia Sanchez
Estela Barraza

APPROVAL: EXPEDITED REVIEW

Gabriel Shaibi
Health Promotion and Disease Prevention, Center for
602/496-0909
Gabriel.Shaibi@asu.edu

Dear Gabriel Shaibi:

On 5/16/2017 the ASU IRB reviewed the following protocol:

Type of Review:	Modification
Title:	Diabetes Prevention for Latino Youth with Prediabetes
Investigator:	Gabriel Shaibi
IRB ID:	STUDY00003735
Category of review:	(mm) Minor modification
Funding:	Name: HHS: National Institutes of Health (NIH), Grant Office ID: 2684, Funding Source ID: 1R01DK107579-01
Grant Title:	None
Grant ID:	None
Documents Reviewed:	• ELSC_Prediabetes_Prescreening_script_clean_3-22-16.pdf, Category: Recruitment Materials;<br>• ELSC Prediabetes Flyer Full page St V _04042016.pub, Category: Recruitment Materials;<br>• Hu_CITI_PCH report (Mar 2016).pdf, Category: Other (to reflect anything not captured above);<br>• Preparing for your Health Screening-Weekend_05052016.pdf, Category: Participant materials (specific directions for them);<br>• SE and Diet.pdf, Category: Measures (Survey questions/Interview questions /interview guides/focus group questions);<br>• Completion Report-EH 12-18-12.pdf, Category: Other (to reflect anything not captured above);<br>• PCP Engagement Letter_08-25-16.pdf, Category: Other (to reflect anything not captured above);<br>• Prediabetes FAQs_St.V..pdf, Category: Recruitment Materials;<br>• WQOL InstrumentEnglish_Spanish.pdf, Category: Measures

	(Survey questions/Interview questions /interview guides/focus group questions);<br>• Prediabetes Pre-Screening Form_St.V._Spanish_04112016.pdf, Category: Recruitment Materials;<br>• T2-T3 Results Letter _very abnormal_10-24-16_Bilingual.pdf, Category: Participant materials (specific directions for them);<br>• ACCULTURATION AHIMSA.pdf, Category: Measures (Survey questions/Interview questions /interview guides/focus group questions);<br>• ELSC_FrontSide.pdf, Category: Participant materials (specific directions for them);<br>• Preparing for your Testing Visit_T1-T3_Weekend_05052016.pdf, Category: Participant materials (specific directions for them);<br>• Pubertal Developmental StatusBOYS.pdf, Category: Measures (Survey questions/Interview questions /interview guides/focus group questions);<br>• Prediabetes Project Summary and Eligibility Criteria for Physicians.pdf, Category: Recruitment Materials;<br>• Shaibi - Diabetes Prevention in Latino youth with prediabetes_PROTOCOL_track changes_02232017.docx, Category: IRB Protocol;<br>• Notification_A1cEligibility_ PhoneScript_English_12-19-16.pdf, Category: Recruitment Materials;<br>• Preparing for your Health Screening-Weekday_05052016.pdf, Category: Participant materials (specific directions for them);<br>• SE and PA.pdf, Category: Measures (Survey questions/Interview questions /interview guides/focus group questions);<br>• Screening Phone Script_clean_03-28-16.pdf, Category: Recruitment Materials;<br>• Screening Phone Script_track changes_03-28-16.pdf, Category: Recruitment Materials;<br>• Results Letter for Ineligible_normal_10-24-16_Bilingual.pdf, Category: Participant materials (specific directions for them);<br>• Brief Acculturation Rating Scale for Mexican Americans (ARMSA II).pdf, Category: Measures (Survey questions/Interview questions /interview guides/focus group questions);<br>• Work Attendance_Parents__Spanish_11012016.pdf, Category: Measures (Survey questions/Interview questions /interview guides/focus group questions);<br>• Backtranslation _12-01-16.pdf, Category: Translations;<br>• Results Letter for Ineligible_abnormal_10-24-16_Bilingual.pdf, Category: Participant materials (specific directions for them);<br>• Pimentel CITI 2015.pdf, Category: Other (to reflect anything not captured above);<br>• Tachicardia_parent_Letter_Spanish_03062017.pdf, Category: Participant materials (specific directions for them);

	• Backtranslation _Recruitmentflyer__04042016.pdf, Category: Translations;<br>• 2007 Block Food Screener.pdf, Category: Measures (Survey questions/Interview questions /interview guides/focus group questions);<br>• SS and Diet Friends.pdf, Category: Measures (Survey questions/Interview questions /interview guides/focus group questions);<br>• Pre-diabetes_Assent_trackchanges_02132017.pdf, Category: Consent Form;<br>• Family History of Diabetes English 5-6-13.pdf, Category: Measures (Survey questions/Interview questions /interview guides/focus group questions);<br>• ELSC Pre-Screening Form_clean_03212016.pdf, Category: Recruitment Materials;<br>• Shaibi - Diabetes Prevention in Latino youth with prediabetes_PROTOCOL_clean_02232017.docx, Category: IRB Protocol;<br>• MReason_CITI (2).pdf, Category: Other (to reflect anything not captured above);<br>• Backtranslation _FamilyHxDiabetes_050616.pdf, Category: Translations;<br>• MD-Provider Clearance Letter_SSRI.pdf, Category: Recruitment Materials;<br>• Tachycardia_parent_letter_02272017.pdf, Category: Participant materials (specific directions for them);<br>• Backtranslation_03062017.pdf, Category: Translations;<br>• Demographics_youth_05052016.pdf, Category: Measures (Survey questions/Interview questions /interview guides/focus group questions);<br>• Preparing for your Testing Visit_T1-T3_Weekday_05052016.pdf, Category: Participant materials (specific directions for them);<br>• SOP tachycardia.pdf, Category: Other (to reflect anything not captured above);<br>• Family History of Diabetes Spanish 5-6-13.pdf, Category: Measures (Survey questions/Interview questions /interview guides/focus group questions);<br>• PCP letter-lab results_11-10-16.pdf, Category: Other (to reflect anything not captured above);<br>• Work Attendance_Youth_10-27-16.pdf, Category: Measures (Survey questions/Interview questions /interview guides/focus group questions);<br>• T2-T3 Results Letter_abnormal_10-24-16_Bilingual.pdf, Category: Participant materials (specific directions for them);<br>• Pictorial Body Image Assessment.pdf, Category: Measures (Survey questions/Interview questions /interview guides/focus group

	questions);<br>• Backtranslation _11-10-16.pdf, Category: Translations;<br>• MD Physical Activity Clearance Form.pdf, Category: Recruitment Materials;<br>• Lab Intake Form_05022016.pdf, Category: Measures (Survey questions/Interview questions /interview guides/focus group questions);<br>• Authorization for Use and Disclosure of Protected Health Information_Spanish_03082016.pdf, Category: Other (to reflect anything not captured above);<br>• Contact Information.pdf, Category: Measures (Survey questions/Interview questions /interview guides/focus group questions);<br>• Results Letter for Ineligible_very abnormal_10-24-16_Bilingual.pdf, Category: Participant materials (specific directions for them);<br>• Prediabetes_Parent_Consent_Spanish_track changes_02132017.pdf, Category: Consent Form;<br>• citirefresher_Ricardo_3-14-14.pdf, Category: Other (to reflect anything not captured above);<br>• Pubertal Developmental StatusGIRLS.pdf, Category: Measures (Survey questions/Interview questions /interview guides/focus group questions);<br>• ELSC_BackSide.pdf, Category: Participant materials (specific directions for them);<br>• CITI Maria Silva 11-18-12.pdf, Category: Other (to reflect anything not captured above);<br>• Mexican American Cultural Values Scale.pdf, Category: Measures (Survey questions/Interview questions /interview guides/focus group questions);<br>• Backtranslation _2-13-17.pdf, Category: Translations;<br>• PCP Recruitment Letter to Patients_Bilingual_1212016.pdf, Category: Recruitment Materials;<br>• MGutierrez_CITI.pdf, Category: Other (to reflect anything not captured above);<br>• Work Attendance_Parents_10-27-16.pdf, Category: Measures (Survey questions/Interview questions /interview guides/focus group questions);<br>• Pre-diabetes_Assent_clean_0213207.pdf, Category: Consent Form;<br>• PARQ.pdf, Category: Recruitment Materials;<br>• 3_Day_Physical_Activity_Recall.pdf, Category: Measures (Survey questions/Interview questions /interview guides/focus group questions);<br>• Prediabetes_Parent_Consent_Spanish_clean_02132017.pdf, Category: Consent Form;<br>•

	Prediabetes_Parental_Consent_English_trackchanges_02132017.pdf, Category: Consent Form;<br>• Blood Draw_IV_Site_Care_Instructions_Bilingual_05052016_aea.pdf, Category: Participant materials (specific directions for them);<br>• T2-T3 Results Letter _normal_10-24-16_Bilingual.pdf, Category: Participant materials (specific directions for them);<br>• Authorization for Use and Disclosure of Protected Health Information_English_03082016.pdf, Category: Other (to reflect anything not captured above);<br>• Prediabetes_ASU_Screening_Phone_Script_SPA_04192016.pdf, Category: Recruitment Materials;<br>• YQOL-SF.pdf, Category: Measures (Survey questions/Interview questions /interview guides/focus group questions);<br>• Authorization for Use of Protected Health Information_03102016.pdf, Category: Translations;<br>• Translation Certificate_05062016.pdf, Category: Translations;<br>• LVeleta_CITI.pdf, Category: Other (to reflect anything not captured above);<br>• Preventing Diabetes in Latino Youth - Submitted.pdf, Category: Sponsor Attachment;<br>• Demographics_adult.pdf, Category: Measures (Survey questions/Interview questions /interview guides/focus group questions);<br>• SS and PA Family and Friends.pdf, Category: Measures (Survey questions/Interview questions /interview guides/focus group questions);<br>• SS and Diet Family.pdf, Category: Measures (Survey questions/Interview questions /interview guides/focus group questions);<br>• Prediabetes_Parental_Consent_English_clean_02132017.pdf, Category: Consent Form;<br>• Rosenberg Self-Esteem Inventory.pdf, Category: Measures (Survey questions/Interview questions /interview guides/focus group questions);<br>• FAQs for Providers_08252016.pdf, Category: Other (to reflect anything not captured above);

The IRB approved the protocol from 1/11/2017 to 1/18/2018 inclusive. Three weeks before 1/18/2018 you are to submit a completed Continuing Review application and required attachments to request continuing approval or closure.

If continuing review approval is not granted before the expiration date of 1/18/2018 approval of this protocol expires on that date. When consent is appropriate, you must use final, watermarked versions available under the “Documents” tab in ERA-IRB.

In conducting this protocol you are required to follow the requirements listed in the INVESTIGATOR MANUAL (HRP-103).

Sincerely,

IRB Administrator

cc: Allison Williams
Crystal Ramos
Allison Williams
Virginia Boyd
Rachel Hernandez
Tatianna Alvarado
Erica Soltero
Inty Moreno
Stephanie Ayers
Samantha Mendez
Janet McNicol
Jessica Reyes
Janae Degroot
Anaid Gonzalvez
Leopoldo Hartmann Manrique
Felipe Castro
Margarita Stirk
Elvia Madrid
Arlene Ramos
Ana Renteria Mexia
Zari Chacon
Colleen Keller
Claudia Sanchez
Estela Barraza

APPROVAL:CONTINUATION

Gabriel Shaibi
Health Promotion and Disease Prevention, Center for
602/496-0909
Gabriel.Shaibi@asu.edu

Dear Gabriel Shaibi:

On 12/13/2017 the ASU IRB reviewed the following protocol:

Type of Review:	Modification and Continuing Review
Title:	Diabetes Prevention for Latino Youth with Prediabetes
Investigator:	Gabriel Shaibi
IRB ID:	STUDY00003735
Category of review:	
Funding:	Name: HHS: National Institutes of Health (NIH), Grant Office ID: 2684, Funding Source ID: 1R01DK107579-01
Grant Title:	None
Grant ID:	None
Documents Reviewed:	• ELSC_Prediabetes_Prescreening_script_clean_3-22-16.pdf, Category: Recruitment Materials;<br>• ELSC Prediabetes Flyer Full page St V _04042016.pub, Category: Recruitment Materials;<br>• Hu_CITI_PCH report (Mar 2016).pdf, Category: Other (to reflect anything not captured above);<br>• Preparing for your Health Screening-Weekend_05052016.pdf, Category: Participant materials (specific directions for them);<br>• SE and Diet.pdf, Category: Measures (Survey questions/Interview questions /interview guides/focus group questions);<br>• Completion Report-EH 12-18-12.pdf, Category: Other (to reflect anything not captured above);<br>• PCP Engagement Letter_08-25-16.pdf, Category: Other (to reflect anything not captured above);<br>• Prediabetes FAQs_St.V..pdf, Category: Recruitment Materials;

	• WQOL InstrumentEnglish_Spanish.pdf, Category: Measures (Survey questions/Interview questions /interview guides/focus group questions);<br>• Prediabetes Pre-Screening Form_St.V._Spanish_04112016.pdf, Category: Recruitment Materials;<br>• T2-T3 Results Letter _very abnormal_10-24-16_Bilingual.pdf, Category: Participant materials (specific directions for them);<br>• ACCULTURATION AHIMSA.pdf, Category: Measures (Survey questions/Interview questions /interview guides/focus group questions);<br>• ELSC_FrontSide.pdf, Category: Participant materials (specific directions for them);<br>• Preparing for your Testing Visit_T1-T3_Weekend_05052016.pdf, Category: Participant materials (specific directions for them);<br>• Pubertal Developmental StatusBOYS.pdf, Category: Measures (Survey questions/Interview questions /interview guides/focus group questions);<br>• Prediabetes_Parent_Consent_Spanish_track_12192017.pdf, Category: Consent Form;<br>• Prediabetes Project Summary and Eligibility Criteria for Physicians.pdf, Category: Recruitment Materials;<br>• Shaibi - Diabetes Prevention in Latino youth with prediabetes_PROTOCOL_track changes_02232017.docx, Category: IRB Protocol;<br>• IRB Memo 1-2-18.pdf, Category: Other (to reflect anything not captured above);<br>• Notification_A1cEligibility_ PhoneScript_English_12-19-16.pdf, Category: Recruitment Materials;<br>• Preparing for your Health Screening-Weekday_05052016.pdf, Category: Participant materials (specific directions for them);<br>• SE and PA.pdf, Category: Measures (Survey questions/Interview questions /interview guides/focus group questions);<br>• Screening Phone Script_clean_03-28-16.pdf, Category: Recruitment Materials;<br>• Screening Phone Script_track changes_03-28-16.pdf, Category: Recruitment Materials;<br>• Results Letter for Ineligible_normal_10-24-16_Bilingual.pdf, Category: Participant materials (specific directions for them);<br>• Brief Acculturation Rating Scale for Mexican Americans (ARMSA II).pdf, Category: Measures (Survey questions/Interview questions /interview guides/focus group questions);<br>• Work Attendance_Parents__Spanish_11012016.pdf, Category: Measures (Survey questions/Interview questions /interview

	guides/focus group questions);<br>• Backtranslation _12-01-16.pdf, Category: Translations;<br>• Results Letter for Ineligible_abnormal_10-24-16_Bilingual.pdf, Category: Participant materials (specific directions for them);<br>• Pimentel CITI 2015.pdf, Category: Other (to reflect anything not captured above);<br>• Tachicardia_parent_Letter_Spanish_03062017.pdf, Category: Participant materials (specific directions for them);<br>• Backtranslation _Recruitmentflyer__04042016.pdf, Category: Translations;<br>• 2007 Block Food Screener.pdf, Category: Measures (Survey questions/Interview questions /interview guides/focus group questions);<br>• SS and Diet Friends.pdf, Category: Measures (Survey questions/Interview questions /interview guides/focus group questions);<br>• Pre-diabetes_Assent_trackchanges_02132017.pdf, Category: Consent Form;<br>• Family History of Diabetes English 5-6-13.pdf, Category: Measures (Survey questions/Interview questions /interview guides/focus group questions);<br>• ELSC Pre-Screening Form_clean_03212016.pdf, Category: Recruitment Materials;<br>• Shaibi - Diabetes Prevention in Latino youth with prediabetes_PROTOCOL_clean_02232017.docx, Category: IRB Protocol;<br>• MReason_CITI (2).pdf, Category: Other (to reflect anything not captured above);<br>• Backtranslation _FamilyHxDiabetes_050616.pdf, Category: Translations;<br>• MD-Provider Clearance Letter_SSRI.pdf, Category: Recruitment Materials;<br>• Tachycardia_parent_letter_02272017.pdf, Category: Participant materials (specific directions for them);<br>• Backtranslation_03062017.pdf, Category: Translations;<br>• Demographics_youth_05052016.pdf, Category: Measures (Survey questions/Interview questions /interview guides/focus group questions);<br>• Preparing for your Testing Visit_T1-T3_Weekday_05052016.pdf, Category: Participant materials (specific directions for them);<br>• SOP tachycardia.pdf, Category: Other (to reflect anything not captured above);<br>• Family History of Diabetes Spanish 5-6-13.pdf, Category: Measures (Survey questions/Interview questions /interview

	guides/focus group questions);<br>• PCP letter-lab results_11-10-16.pdf, Category: Other (to reflect anything not captured above);<br>• Work Attendance_Youth_10-27-16.pdf, Category: Measures (Survey questions/Interview questions /interview guides/focus group questions);<br>• T2-T3 Results Letter_abnormal_10-24-16_Bilingual.pdf, Category: Participant materials (specific directions for them);<br>• Pictorial Body Image Assessment.pdf, Category: Measures (Survey questions/Interview questions /interview guides/focus group questions);<br>• Backtranslation _11-10-16.pdf, Category: Translations;<br>• MD Physical Activity Clearance Form.pdf, Category: Recruitment Materials;<br>• Lab Intake Form_05022016.pdf, Category: Measures (Survey questions/Interview questions /interview guides/focus group questions);<br>• Authorization for Use and Disclosure of Protected Health Information_Spanish_03082016.pdf, Category: Other (to reflect anything not captured above);<br>• Contact Information.pdf, Category: Measures (Survey questions/Interview questions /interview guides/focus group questions);<br>• Prediabetes_Parental_Consent_English_track_12192017.pdf, Category: Consent Form;<br>• Pre-diabetes_Assent_clean_12-19-177.pdf, Category: Consent Form;<br>• Prediabetes_Parental_Consent_English_clean_12192017.pdf, Category: Consent Form;<br>• Results Letter for Ineligible_very abnormal_10-24-16_Bilingual.pdf, Category: Participant materials (specific directions for them);<br>• citirefresher_Ricardo_3-14-14.pdf, Category: Other (to reflect anything not captured above);<br>• Pubertal Developmental StatusGIRLS.pdf, Category: Measures (Survey questions/Interview questions /interview guides/focus group questions);<br>• ELSC_BackSide.pdf, Category: Participant materials (specific directions for them);<br>• CITI Maria Silva 11-18-12.pdf, Category: Other (to reflect anything not captured above);<br>• Mexican American Cultural Values Scale.pdf, Category: Measures (Survey questions/Interview questions /interview guides/focus group questions);<br>• Backtranslation _2-13-17.pdf, Category: Translations;<br>• PCP Recruitment Letter to Patients_Bilingual_1212016.pdf,

	Category: Recruitment Materials;<br>• MGutierrez_CITI.pdf, Category: Other (to reflect anything not captured above);<br>• Work Attendance_Parents_10-27-16.pdf, Category: Measures (Survey questions/Interview questions /interview guides/focus group questions);<br>• Pre-diabetes_Assent_clean_0213207.pdf, Category: Consent Form;<br>• PARQ.pdf, Category: Recruitment Materials;<br>• 3_Day_Physical_Activity_Recall.pdf, Category: Measures (Survey questions/Interview questions /interview guides/focus group questions);<br>• Prediabetes_Parent_Consent_Spanish_clean_12192017.pdf, Category: Consent Form;<br>• Blood Draw_IV_Site_Care_Instructions_Bilingual_05052016_aea.pdf, Category: Participant materials (specific directions for them);<br>• T2-T3 Results Letter _normal_10-24-16_Bilingual.pdf, Category: Participant materials (specific directions for them);<br>• Authorization for Use and Disclosure of Protected Health Information_English_03082016.pdf, Category: Other (to reflect anything not captured above);<br>• Prediabetes_ASU_Screening_Phone_Script_SPA_04192016.pdf, Category: Recruitment Materials;<br>• YQOL-SF.pdf, Category: Measures (Survey questions/Interview questions /interview guides/focus group questions);<br>• Authorization for Use of Protected Health Information_03102016.pdf, Category: Translations;<br>• Translation Certificate_05062016.pdf, Category: Translations;<br>• LVeleta_CITI.pdf, Category: Other (to reflect anything not captured above);<br>• Preventing Diabetes in Latino Youth - Submitted.pdf, Category: Sponsor Attachment;<br>• Demographics_adult.pdf, Category: Measures (Survey questions/Interview questions /interview guides/focus group questions);<br>• SS and PA Family and Friends.pdf, Category: Measures (Survey questions/Interview questions /interview guides/focus group questions);<br>• SS and Diet Family.pdf, Category: Measures (Survey questions/Interview questions /interview guides/focus group questions);<br>• Rosenberg Self-Esteem Inventory.pdf, Category: Measures (Survey questions/Interview questions /interview guides/focus

	group questions);<br>• FAQs for Providers_08252016.pdf, Category: Other (to reflect anything not captured above);

The IRB approved the protocol from 12/13/2017 to 12/12/2018 inclusive. Three weeks before 12/12/2018 you are to submit a completed Continuing Review application and required attachments to request continuing approval or closure.

If continuing review approval is not granted before the expiration date of 12/12/2018 approval of this protocol expires on that date. When consent is appropriate, you must use final, watermarked versions available under the "Documents" tab in ERA-IRB.

In conducting this protocol you are required to follow the requirements listed in the INVESTIGATOR MANUAL (HRP-103).

Sincerely,

IRB Administrator

cc: Allison Williams
Elizabeth Biggs
Colleen Keller
Erica Soltero
Janet McNicol
Neeku Navabi
Arlene Ramos
Tatianna Alvarado
Shannon McCarthy
Ana Renteria Mexia
Jared Treichel
Stephanie Ayers
Rachel Hernandez
Claudia Sanchez
Zari Chacon
Jessica Camacho
Erica Basco
Elvia Madrid
Felipe Castro
Virginia Boyd
Samantha Mendez
Allison Williams
Armando Pena
Leopoldo Hartmann Manrique
Anaid Gonzalvez

Jessica Reyes
Janae Degroot
Rhiannon Worker
Crystal Ramos

APPROVAL: MODIFICATION

Gabriel Shaibi
EDSON: Health Promotion and Disease Prevention, Center for
602/496-0909
Gabriel.Shaibi@asu.edu

Dear Gabriel Shaibi:

On 10/3/2019 the ASU IRB reviewed the following protocol:

Type of Review:	Modification/Update
Title:	Diabetes Prevention for Latino Youth with Prediabetes
Investigator:	Gabriel Shaibi
IRB ID:	STUDY00003735
Funding:	Name: HHS: National Institutes of Health (NIH), Grant Office ID: 2684, Funding Source ID: 1R01DK107579-01; Name: American Heart Association, Grant Office ID: FP00013949
Grant Title:	None
Grant ID:	None
Documents Reviewed:	• Backtranslation-ScreeningQuestion-GroupSetting, Category: Screening forms;<br>• ASU Screening Form_09302019.pdf, Category: Screening forms;<br>• Shaibi - Diabetes Prevention in Latino youth with prediabetes_PROTOCOL_clean_04162019.docx, Category: IRB Protocol;

The IRB approved the modification.

When consent is appropriate, you must use final, watermarked versions available under the “Documents” tab in ERA-IRB.

In conducting this protocol you are required to follow the requirements listed in the INVESTIGATOR MANUAL (HRP-103).

Sincerely,

IRB Administrator

cc: Arlene Fernandez
Elvia Madrid
Siddhartha Angadi
Sebastian Delarosa
Tiara Khalid
Jared Treichel
Kristin Shea
Dania Alcala-Calvillo
Veronica Zamora
Stavros Kavouras
Abigail Colburn
Ana Martinez Valencia
Paulina Arce
Erica Soltero
Hanna Rahman
Shannon McCarthy
Leopoldo Hartmann Manrique
Neeku Navabi
Alana Herman
Arlene Fernandez
Rhiannon Worker
Janae Degroot
Inty Moreno
Jean Figueroa
HyunGyu Suh
Felipe Castro
Ana Renteria Mexia
Jessica Camacho
Stephanie Ayers
Mariana Rodriguez
Armando Pena
Kiley Vander Wyst
Allison Williams
Amanda Campos
Tatianna Alvarado
Daniel Munoz
Virginia Boyd
Samantha Mendez
Isaac Carranza
Lourdes Estrada

APPROVAL: MODIFICATION

Gabriel Shaibi
EDSON: Health Promotion and Disease Prevention, Center for
602/496-0909
Gabriel.Shaibi@asu.edu

Dear Gabriel Shaibi:

On 12/30/2019 the ASU IRB reviewed the following protocol:

Type of Review:	Modification / Update
Title:	Diabetes Prevention for Latino Youth with Prediabetes
Investigator:	Gabriel Shaibi
IRB ID:	STUDY00003735
Funding:	Name: HHS: National Institutes of Health (NIH), Grant Office ID: 2684, Funding Source ID: 1R01DK107579-01; Name: American Heart Association, Grant Office ID: FP00013949
Grant Title:	None
Grant ID:	None
Documents Reviewed:	• ELSC KeyInformantInterviewProtocol_12302019.pdf, Category: Measures (Survey questions/Interview questions /interview guides/focus group questions);<br>• KeyInformantConsent-InterviewPhone_010320doc.pdf, Category: Consent Form;<br>• KeyInformantConsent-Past Participant_010320.pdf, Category: Consent Form;<br>• Script-Key Informants_12302019.pdf, Category: Recruitment Materials;<br>• Shaibi-Diabetes Prevention in Latino youth with prediabetes_PROTOCOL_clean_010320.docx, Category: IRB Protocol;

The IRB approved the modification.

When consent is appropriate, you must use final, watermarked versions available under the “Documents” tab in ERA-IRB.

In conducting this protocol you are required to follow the requirements listed in the INVESTIGATOR MANUAL (HRP-103).

Sincerely,

IRB Administrator

cc: Arlene Fernandez
Isaac Carranza
Kristin Shea
Jared Treichel
Arlene Fernandez
Siddhartha Angadi
Jessica Camacho
Stavros Kavouras
Veronica Zamora
Samantha Mendez
Rhiannon Worker
Hanna Rahman
Virginia Boyd
Tiara Khalid
Dania Alcala-Calvillo
James Sandoval
Shannon McCarthy
Stephanie Ayers
Lourdes Estrada
Leonel Lopez
Alana Herman
Paulina Arce
Tatianna Alvarado
HyunGyu Suh
Neeku Navabi
Janae Degroot
Inty Moreno
Amanda Campos
Kiley Vander Wyst
Erica Soltero
Leopoldo Hartmann Manrique
Sebastian Delarosa
Armando Pena

Ana Martinez Valencia
Jean Figueroa
Ana Renteria Mexia
Mariana Rodriguez
Elvia Madrid
Daniel Munoz
Macy Wright
Abigail Colburn
Allison Williams
Felipe Castro

APPROVAL: MODIFICATION

Gabriel Shaibi
EDSON: Health Promotion and Disease Prevention, Center for
602/496-0909
Gabriel.Shaibi@asu.edu

Dear Gabriel Shaibi:

On 10/6/2020 the ASU IRB reviewed the following protocol:

Type of Review:	Modification / Update
Title:	Diabetes Prevention for Latino Youth with Prediabetes
Investigator:	Gabriel Shaibi
IRB ID:	STUDY00003735
Funding:	Name: HHS: National Institutes of Health (NIH), Grant Office ID: 2684, Funding Source ID: 1R01DK107579-01; Name: HHS: National Institutes of Health (NIH), Grant Office ID: FP00021180_Res1, Funding Source ID: 1 F31 DK125037-01A1; Name: American Heart Association, Grant Office ID: FP00013949
Grant Title:	None
Grant ID:	None
Documents Reviewed:	None

The IRB approved the modification.

When consent is appropriate, you must use final, watermarked versions available under the “Documents” tab in ERA-IRB.

In conducting this protocol you are required to follow the requirements listed in the INVESTIGATOR MANUAL (HRP-103).

Sincerely,

IRB Administrator

cc: Allison Williams
Leonel Lopez
Dania Alcala-Calvillo
Veronica Zamora
Elvia Madrid
Kristin Shea
Amanda Campos
Ana Renteria Mexia
Jared Treichel
Hyun-Gyu Suh
Shannon McCarthy
Stavros Kavouras
James Sandoval
Daniel Munoz
Tatianna Alvarado
Neeku Navabi
Janae Degroot
Macy Wright
Alana Herman
Allison Williams
Lourdes Estrada
Inty Moreno
Tiara Khalid
Kiley Vander Wyst
Arlene Fernandez
Ana Martinez Valencia
Jean Figueroa
Mariana Rodriguez
Jessica Camacho
Abigail Colburn
Stephanie Ayers
Armando Pena
Hanna Rahman
Samantha Mendez
Sebastian Delarosa
Erica Soltero
Rhiannon Worker
Felipe Castro
Siddhartha Angadi
Paulina Arce
Leopoldo Hartmann Manrique
Isaac Carranza

APPROVAL: MODIFICATION

Gabriel Shaibi
EDSON: Health Promotion and Disease Prevention, Center for
602/496-0909
Gabriel.Shaibi@asu.edu

Dear Gabriel Shaibi:

On 1/14/2021 the ASU IRB reviewed the following protocol:

Type of Review:	Modification / Update
Title:	Diabetes Prevention for Latino Youth with Prediabetes
Investigator:	Gabriel Shaibi
IRB ID:	STUDY00003735
Funding:	Name: HHS: National Institutes of Health (NIH), Grant Office ID: 2684, Funding Source ID: 1R01DK107579-01; Name: American Heart Association, Grant Office ID: FP00013949; Name: HHS: National Institutes of Health (NIH), Grant Office ID: FP00021180_Res1, Funding Source ID: 1 F31 DK125037-01A1
Grant Title:	None
Grant ID:	None
Documents Reviewed:	• ASU MOD00001245 to MTA0567_Shaibi_FE.pdf, Category: Other;

The IRB approved the modification.

When consent is appropriate, you must use final, watermarked versions available under the “Documents” tab in ERA-IRB.

In conducting this protocol you are required to follow the requirements listed in the INVESTIGATOR MANUAL (HRP-103).

Sincerely,

IRB Administrator

cc: Allison Williams
Ana Martinez Valencia
Stavros Kavouras
Hyun-Gyu Suh
Erica Soltero
Veronica Zamora
Allison Williams
Elvia Madrid
Arlene Fernandez
Kiley Vander Wyst
Armando Pena
Sebastian Delarosa
Dania Alcala-Calvillo
Abigail Colburn
Felipe Castro
James Sandoval
Stephanie Ayers
Lourdes Estrada

ASU Knowledge Enterprise Development

APPROVAL: MODIFICATION

Gabriel Shaibi
EDSON: Health Promotion and Disease Prevention, Center for
602/496-0909
Gabriel.Shaibi@asu.edu

Dear Gabriel Shaibi:

On 4/6/2021 the ASU IRB reviewed the following protocol:

Type of Review:	Modification / Update
Title:	Diabetes Prevention for Latino Youth with Prediabetes
Investigator:	Gabriel Shaibi
IRB ID:	STUDY00003735
Funding:	Name: Arizona State University (ASU); Name: HHS: National Institutes of Health (NIH), Grant Office ID: 2684, Funding Source ID: 1R01DK107579-01; Name: American Heart Association, Grant Office ID: FP00013949; Name: HHS: National Institutes of Health (NIH), Grant Office ID: FP00021180_Res1, Funding Source ID: 1 F31 DK125037-01A1
Grant Title:	None
Grant ID:	None
Documents Reviewed:	• ASU_ISSR_AwardLetter, Category: Sponsor Attachment;<br>• ISSR application_FINAL.docx, Category: Sponsor Attachment;<br>• ISSR application_FINAL.pdf, Category: Other;<br>• ISSR Interview Questions_04022021.pdf, Category: Other;<br>• ISSR_AwardLetter, Category: Other;<br>• ISSR_InformedConsent_04022021.pdf, Category: Consent Form;<br>• Shaibi-Diabetes Prevention in Latino youth with prediabetes_PROTOCOL_clean_03312021.docx, Category: IRB Protocol;

	• Shaibi-Diabetes Prevention in Latino youth with prediabetes_PROTOCOL_tracked_03312021.docx, Category: IRB Protocol;

The IRB approved the modification.

When consent is appropriate, you must use final, watermarked versions available under the “Documents” tab in ERA-IRB.

In conducting this protocol you are required to follow the requirements listed in the INVESTIGATOR MANUAL (HRP-103).

Sincerely,

IRB Administrator

cc: Arlene Fernandez
Ana Martinez Valencia
Armando Pena
Abigail Colburn
Elvia Madrid
Stephanie Ayers
Allison Williams
Veronica Zamora
Dania Alcala-Calvillo
Arlene Fernandez
Sebastian Delarosa
Kiley Vander Wyst
Erica Soltero
Hyun-Gyu Suh
Ismail Alvarado
Felipe Castro
Stavros Kavouras

APPROVAL: MODIFICATION

Gabriel Shaibi
EDSON: Health Promotion and Disease Prevention, Center for
602/496-0909
Gabriel.Shaibi@asu.edu

Dear Gabriel Shaibi:

On 5/15/2021 the ASU IRB reviewed the following protocol:

Type of Review:	Modification / Update
Title:	Diabetes Prevention for Latino Youth with Prediabetes
Investigator:	Gabriel Shaibi
IRB ID:	STUDY00003735
Funding:	Name: Arizona State University (ASU); Name: HHS: National Institutes of Health (NIH), Grant Office ID: 2684, Funding Source ID: 1R01DK107579-01; Name: American Heart Association, Grant Office ID: FP00013949; Name: HHS: National Institutes of Health (NIH), Grant Office ID: FP00021180_Res1, Funding Source ID: 1 F31 DK125037-01A1
Grant Title:	None
Grant ID:	None
Documents Reviewed:	• ISSR_RevisedProtocol_Version3_05182021.pdf, Category: Other;

The IRB approved the modification.

When consent is appropriate, you must use final, watermarked versions available under the "Documents" tab in ERA-IRB.

In conducting this protocol you are required to follow the requirements listed in the INVESTIGATOR MANUAL (HRP-103).

Sincerely,

IRB Administrator

cc: Arlene Fernandez
Ana Martinez Valencia
Armando Pena
Abigail Colburn
Elvia Madrid
Stephanie Ayers
Allison Williams
Veronica Zamora
Dania Alcala-Calvillo
Arlene Fernandez
Sebastian Delarosa
Kiley Vander Wyst
Erica Soltero
Hyun-Gyu Suh
Ismail Alvarado
Felipe Castro
Stavros Kavouras

APPROVAL: MODIFICATION

Gabriel Shaibi
EDSON: Health Promotion and Disease Prevention, Center for
602/496-0909
Gabriel.Shaibi@asu.edu

Dear Gabriel Shaibi:

On 2/21/2022 the ASU IRB reviewed the following protocol:

Type of Review:	Modification / Update
Title:	Diabetes Prevention for Latino Youth with Prediabetes
Investigator:	Gabriel Shaibi
IRB ID:	STUDY00003735
Funding:	Name: HHS: National Institutes of Health (NIH), Grant Office ID: 2684, Funding Source ID: 1R01DK107579-01; Name: Arizona State University (ASU); Name: American Heart Association, Grant Office ID: FP00013949; Name: HHS: National Institutes of Health (NIH), Grant Office ID: FP00021180_Res1, Funding Source ID: 1 F31 DK125037-01A1
Grant Title:	None
Grant ID:	None
Documents Reviewed:	None

The IRB approved the modification.

When consent is appropriate, you must use final, watermarked versions available under the “Documents” tab in ERA-IRB.

In conducting this protocol you are required to follow the requirements listed in the INVESTIGATOR MANUAL (HRP-103).

REMINDER – Effective January 12th 2022, in-person interactions with human subjects require adherence to all current policies for ASU faculty, staff, students and visitors. Up-

to-date information regarding ASU's COVID-19 Management Strategy can be found here. IRB approval is related to the research activity involving human subjects, all other protocols related to COVID-19 management including face coverings, health checks, facility access, etc. are governed by current ASU policy.

Sincerely,

IRB Administrator

cc: Allison Williams
Dania Alcala-Calvillo
Allison Williams
Abigail Colburn
Stavros Kavouras
Stephanie Ayers
Kiley Vander Wyst
Arlene Fernandez
Ismail Alvarado
Felipe Castro
Armando Pena
