# Supplementary material for: Effects of a Diabetes Prevention Program on Type 2 Diabetes Risk Factors and Quality of Life Among Latino Youths With Prediabetes: A Randomized Clinical Trial
Source: JAMA Netw Open. 2022 Sep 12;5(9):e2231196. doi: 10.1001/jamanetworkopen.2022.31196 (PMC9468887; doi:10.1001/jamanetworkopen.2022.31196)
Supplement: Supplement 2. — eTable 1. Baseline Sociodemographic Data for All Youth eTable 2. Baseline Sociodemographic Data as Reported by Parents eTable 3. Changes in Physical Activity Within and Between Groups [file jamanetwopen-e2231196-s002.pdf]

# Supplemental Online Content

Peña A, Olson ML, Hooker E, et al. Effects of a diabetes prevention program on type 2 diabetes risk factors and quality of life among Latino youths with prediabetes: a randomized clinical trial. *JAMA Netw Open.* 2022;5(9):e2231196. doi:10.1001/jamanetworkopen.2022.31196

**eTable 1.** Baseline Sociodemographic Data for All Youth

**eTable 2.** Baseline Sociodemographic Data as Reported by Parents

**eTable 3.** Changes in Physical Activity Within and Between Groups

This supplemental material has been provided by the authors to give readers additional information about their work.

© 2022 Peña A et al. *JAMA Network Open.*

| **eTable 1**. Baseline Sociodemographic Data for All Youth (n=117) | | | |
|---|---|---|---|
| | | **UCC (n=38)** | **INT (n=79)** |
| Parameter | | n (%) | n (%) |
| Ethnicity | *Mexican* | 33 (87) | 76 (96) |
| | *Central American* | 3 (7) | 1 (12) |
| | *South American* | 1 (3) | 0 (0) |
| | *Mexican/Central American* | 1 (3) | 2 (2) |
| | | | |
| Country of Origin | *United States* | 26 (68) | 58 (73) |
| | *Mexico* | 3 (8) | 8 (10) |
| | *Central America* | 1 (3) | 0 (0) |
| | *Did not respond* | 8 (21) | 13 (17) |
| | | | |
| Preferred Language | *English* | 29 (76) | 67 (85) |
| | *Spanish* | 6 (16) | 7 (9) |
| | *Did not respond* | 3 (8) | 5 (6) |

| **eTable 2**. Baseline Sociodemographic Data as Reported by Parents (n=117) | | | |
|---|---|---|---|
| | | **UCC (n=38)** | **INT (n=79)** |
| Parameter | | n (%) | n (%) |
| Monthly Income | *$0 - 500* | 10 (26) | 7 (9) |
| | *$501 - 1,000* | 10 (26) | 19 (24) |
| | *$1,001 - 2,000* | 11 (29) | 22 (28) |
| | *$2,001 - 3,000* | 3 (8) | 13 (16) |
| | *$3,001 - 4,000* | 1 (2) | 7 (9) |
| | *$4,001 - 5,000* | 0 (0) | 4 (5) |
| | *Other amount* | 0 (0) | 2 (3) |
| | *Do not know* | 1 (2) | 3 (4) |
| | *Refused to respond* | 1 (2) | 1 (1) |
| | *Did not respond* | 2 (5) | 1 (1) |
| | | | |
| Government Assistance Programs | *WIC Services* | 1 (3) | 2 (3) |
| | *Medicaid* | 8 (21) | 30 (38) |
| | *Food Stamps* | 2 (5) | 1 (1) |
| | *WIC Services/Medicaid* | 4 (11) | 5 (6) |
| | *WIC/Food Stamps* | 0 (0) | 0 (0) |
| | *Medicaid/Food Stamps* | 7 (18) | 21 (26) |
| | *WIC/Medicaid/Food Stamps* | 3 (8) | 10 (13) |
| | *None* | 13 (34) | 10 (13) |
| WIC: Special Supplemental Nutrition Program for Women, Infant, and Child | | | |

© 2022 Peña A et al. *JAMA Network Open.*

| **eTable 3**. Changes in Physical Activity Within and Between Groups | | | | | | | | | | |
|---|---|---|---|---|---|---|---|---|---|---|
| | **Usual Care Control** | | | Within-group | **Lifestyle Intervention** | | | Within-group | Treatment Effects | |
| Parameter | *n* | T1 Mean (SE), min/d | T2 Mean (SE), min/d | T2-T1 p-value | *n* | T1 Mean (SE), min/d | T2 Mean (SE), min/d | T2-T1 p-value | ΔT2-T1 (95%CI) | p-value |
| MVPA | 38 | 61 (10) | 55 (11) | 0.67 | 79 | 64 (7) | 60 (8) | p=0.68 | -2 (-39, 35) | 0.91 |
| MVPA: moderate-vigorous physical activity | | | | | | | | | | |

© 2022 Peña A et al. *JAMA Network Open*.
