# Supplementary material for: Effects of a Diabetes Prevention Program on Type 2 Diabetes Risk Factors and Quality of Life Among Latino Youths With Prediabetes: A Randomized Clinical Trial
Source: JAMA Netw Open. 2022 Sep 12;5(9):e2231196. doi: 10.1001/jamanetworkopen.2022.31196 (PMC9468887; doi:10.1001/jamanetworkopen.2022.31196)
Supplement: Supplement 3. — Data Sharing Statement [file jamanetwopen-e2231196-s003.pdf]

Data Sharing Statement

Peña. Effects of a Diabetes Prevention Program on Type 2 Diabetes Risk Factors and Quality of Life Among Latino Youths With Prediabetes. *JAMA Netw Open*. Published September 12, 2022. doi:10.1001/jamanetworkopen.2022.31196

Data
Data available: No

Additional Information
Explanation for why data not available: This was not included in our consents or IRB protocol
